# Elucidation of the Electrocatalytic Nitrite Reduction Mechanism by Bio-Inspired Copper Complexes

**DOI:** 10.1021/acscatal.3c01989

**Published:** 2023-07-18

**Authors:** Phebe H. van Langevelde, Silène Engbers, Francesco Buda, Dennis G. H. Hetterscheid

**Affiliations:** Leiden Institute of Chemistry, Leiden University, 2300 RA Leiden, The Netherlands

**Keywords:** nitrite reduction, bio-inspired catalysis, electrocatalysis, general acid catalysis, copper complexes

## Abstract

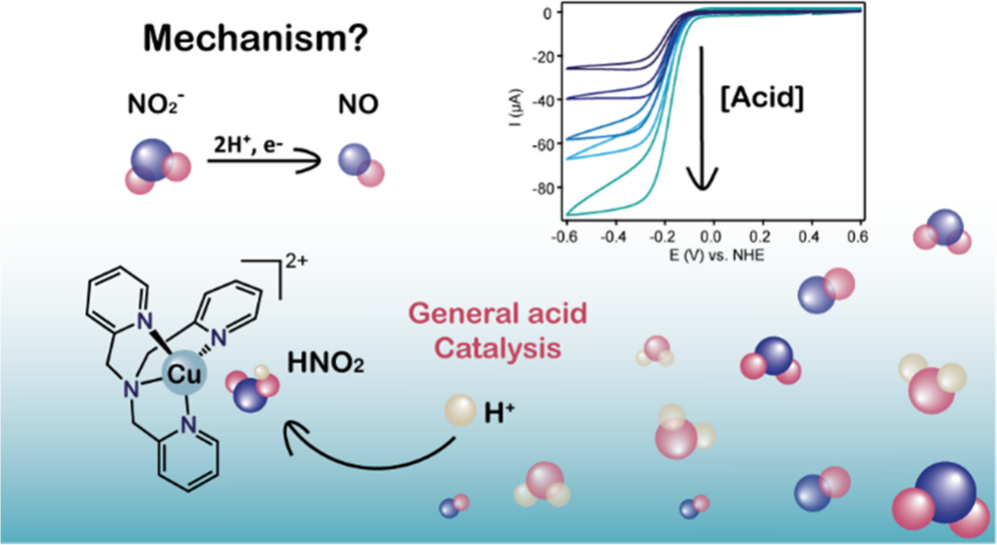

Mononuclear copper complexes relevant to the active site of copper nitrite reductases (CuNiRs) are known to be catalytically active for the reduction of nitrite. Yet, their catalytic mechanism has thus far not been resolved. Here, we provide a complete description of the electrocatalytic nitrite reduction mechanism of a bio-inspired CuNiR catalyst Cu(tmpa) (tmpa = tris(2-pyridylmethyl)amine) in aqueous solution. Through a combination of electrochemical studies, reaction kinetics, and density functional theory (DFT) computations, we show that the protonation steps take place in a stepwise manner and are decoupled from electron transfer. The rate-determining step is a general acid-catalyzed protonation of a copper-ligated nitrous acid (HNO_2_) species. In view of the growing urge to convert nitrogen-containing compounds, this work provides principal reaction parameters for efficient electrochemical nitrite reduction. This contributes to the investigation and development of nitrite reduction catalysts, which is crucial to restore the biogeochemical nitrogen cycle.

## Introduction

The biogeochemical nitrogen flow is the chemical cycle that has most dramatically exceeded the planetary boundary conditions for safe living conditions, given that fixed nitrogen is rapidly accumulating across the globe, leading locally to major problems.^1^ In the global nitrogen cycle, denitrification plays a key role as it is the pathway along which fixed nitrogen is released back into the atmosphere as gaseous N_2_. Denitrification takes place in a wide range of bacteria and archaea as a pathway for anaerobic respiration and entails several reaction steps.^2,3^ Among these steps, the reduction of nitrite (NO_2_^–^) to nitric oxide (NO) is of great interest as NO_2_^–^ and NO are connected to several processes that negatively affect the global nitrogen cycle.^4−7^ For example, the use of nitrogen-rich fertilizers and industrial waste disposal results in increased levels of nitrite and nitrate in groundwater and surface water. In turn, bacteria in the soil can convert nitrite to N_2_O, which is detrimental to the environment.^3^ On the other hand, NO production, albeit harmful to the atmosphere, is attractive for biomedical applications such as NO-releasing catheters^8−14^ and as a reagent in organic synthetic reactions.^15,16^

**Figure 1:**
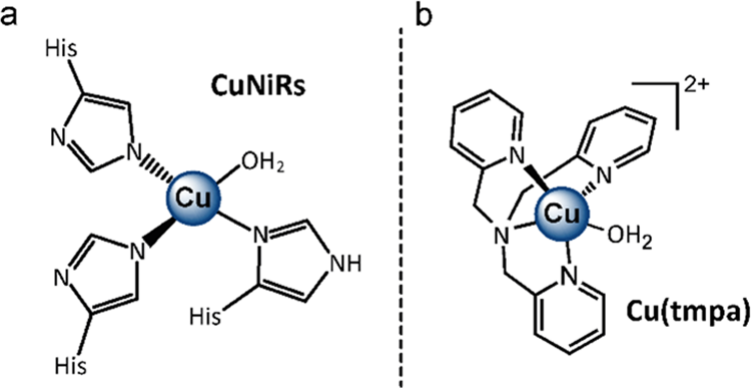
Schematic overview of (a) the mononuclear type 2 copper site in CuNiRs and (b) the Cu(tmpa) catalyst used in this work.

The one-electron reduction of NO_2_^–^ to NO is the second step of the denitrification process and occurs in the metalloenzyme nitrite reductase (NiR), containing either iron or copper in its active site.^17,18^ In CuNiRs, reduction of NO_2_^–^ takes place at a mononuclear type 2 Cu site (Figure 1a).^17,19^ Inspired by the enzymatic process, nitrite reduction is studied using bio-inspired CuNiRs. The reactivity of various Cu(I)^20−30^ and Cu(II)^22,26−29,31−41^ model compounds with nitrite was investigated, and several Cu(II)–NO^27,42−44^ and Cu(I)–NO^45−47^ complexes were characterized. In addition, studies by Fujii and co-workers showed that a stepwise protonation mechanism is operative in dichloromethane,^23,25^ and work by Hsu and co-workers sheds light on the possible formation of HNO_2_ at low pH.^38^

In recent years, bio-inspired CuNiRs are also studied using electrochemical methods in both organic^34,48,49^ and aqueous solutions.^11−14,37,50−52^ The first electrochemical studies on nitrite reduction were reported in 1993 and 1995 by the group of Komeda.^37,52^ Herein, the authors report that [Cu(tmpa)(OH_2_)]^2+^ (tmpa = tris(2-pyridylmethyl)amine, **Cu(tmpa)**, see Figure 1b) reduces NO_2_^–^ electrocatalytically to NO. Later, the Meyerhoff group reported its use in NO-releasing catheters, showing that **Cu(tmpa)** is a stable catalyst for more than 7 days.^11^ Modifications of the **Cu(tmpa)** complex were reported in follow-up studies to improve the Cu(II) reduction potential and faradaic efficiency to NO,^12,13^ to study the role of a proton shuttle in acetonitrile,^48^ or to investigate the catalytic activity by immobilization on a gold surface.^51^

Remarkably, these studies on **Cu(tmpa)** and other bio-inspired CuNiRs did thus far not report which elementary steps are involved in the reaction mechanism. At present, it is not known whether one or both proton transfer steps are decoupled from electron transfer, as is the case in the enzymatic environment of CuNiRs.^53^ Moreover, it is not resolved which of these elementary steps represents the rate-determining step of the nitrite reduction reaction. Up to now, the majority of research has focused on the isolation of catalytic intermediates to determine whether binding of nitrite or nitric oxide occurs via O or N, and to which extent the catalytic activity can be tuned by ligand design. Yet, it has been established that there is a strong pH dependence on the catalytic activity of Cu sites,^11,52^ which is fully to be expected for a reaction with the overall reaction stoichiometry that is required to reduce NO_2_^–^ to NO and H_2_O.

Moreover, recent studies on electrocatalytic nitrite reduction using metals other than copper have addressed the important role of the buffer during catalysis. The group of Smith and co-workers has reported that phosphate buffer assists in the aqueous electrocatalytic reduction of nitrite, making their cobalt-based complex active for the reduction of nitrite.^54^ Likewise, the group of Bren and co-workers reported an iron-based complex for the reduction of nitrite to hydroxylamine and ammonium that is only active in the presence of buffer.^55^

Following this, we report here for the first time a detailed and complete catalytic cycle of the electrocatalytic nitrite reduction mechanism in aqueous solution by a bio-inspired CuNiR catalyst, **Cu(tmpa)**, and pinpoint important reaction parameters for the development and electrochemical analysis of new bio-inspired CuNiR species and other catalytic sites for the nitrite reduction reaction.

## Results and Discussion

**Figure 2:**
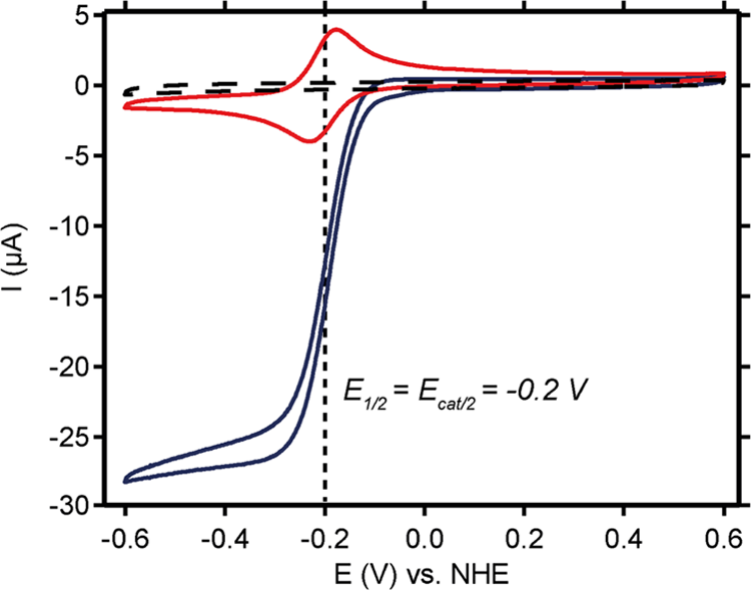
CV measurements of Cu(tmpa) in the absence of substrate (red line) and in the presence of 20 mM NaNO_2_ (blue line) compared to the activity of the GC electrode in the absence of catalyst and in the presence of 20 mM NaNO_2_ (dashed black line). Potential of *E*_1/2_ and *E*_cat/2_ is indicated by a vertical dotted line. Conditions: 0.3 mM Cu(tmpa), Ar atmosphere, 100 mV/s scan rate, 293 K.

Cyclic voltammograms (CVs) of **Cu(tmpa)** were recorded in a 0.1 M phosphate buffer (PB) solution of pH 7, using a glassy carbon (GC) working electrode under an Ar atmosphere (1 atm) (see Figure 2). In the absence of substrate, the reversible Cu^II^/Cu^I^ redox couple of the catalyst is observed. In the presence of 20 mM NaNO_2_, a plateau-shaped catalytic wave appears of which the onset coincides with the reduction of **Cu(tmpa)**. The GC working electrode does not catalyze the reduction of nitrite; therefore, all catalytic current can be attributed to **Cu(tmpa)**. The S-shape of the catalytic wave indicates that the reaction is under pure kinetic conditions. As a result, the half-wave potential of the Cu^II^/Cu^I^ redox couple (*E*_1/2_) lies close to the potential where half of the catalytic current is attained (*E*_cat/2_), being 0.208 and 0.219 V vs RHE, respectively. Repetition of these experiments with forced convection of the solution using a rotating disk electrode (RDE) setup shows that the plateau-current is independent of the rotation speed, which also affirms that the reaction is under kinetic control (see Figure S1). The homogeneity of the catalyst was investigated in additional CV experiments. These measurements confirm that the complete catalytic current originates from the homogeneous catalyst in solution and that **Cu(tmpa)** does not form any catalytically active heterogeneous deposits on the electrode during catalysis (see Supporting Information Section 3).

### Reaction Order in NO_2_^–^ and **Cu(tmpa)**

To clarify the catalytic nitrite reduction mechanism, the reaction orders in nitrite and **Cu(tmpa)** were determined. In a previous study on electrocatalytic nitrite reduction by **Cu(tmpa)**, Meyerhoff and co-workers reported that the catalytic current depends on the nitrite concentration, but the reaction order was not extracted from their data.^11^ For determination of the reaction order, we rationalized that **Cu(tmpa)** selectively catalyzes the one-electron reduction of nitrite. It is known that **Cu**^**I**^**(tmpa)** is also able to produce N_2_O during the reduction of nitrite,^11,52^ due to the disproportionation of the generated NO to N_2_O in protic solvents.^56,57^ However, this side reaction does not lead to the consumption of extra electrons and is suppressed in the presence of high concentrations of NO_2_^–^ and low concentrations of NO, as binding of NO to **Cu(tmpa)** is only weak.^11,46^

**Figure 3:**
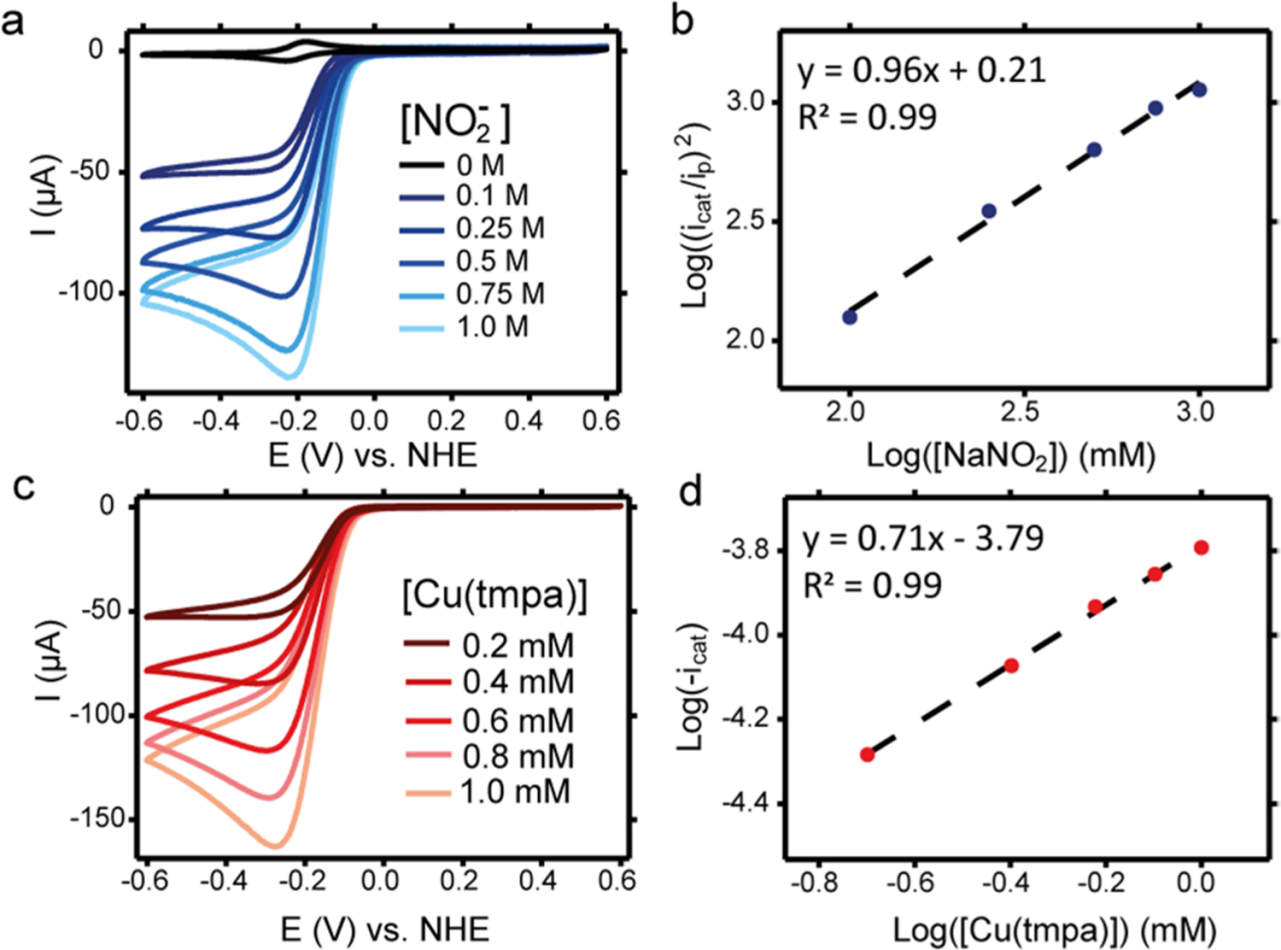
(a) CVs of 0.3 mM Cu(tmpa) in the presence of 0–1.0 M NO_2_^–^ and (b) the corresponding logarithmic plot of (*i*_cat_/*i*_p_)^2^ as a function of [NaNO_2_]; *i*_cat_ is determined as the current at −0.23 V vs NHE. (c) CVs of different Cu(tmpa) concentrations (0.2–1.0 mM) in the presence of 250 mM NaNO_2_ and (d) the corresponding logarithmic plot of −*i*_cat_ as a function of [Cu(tmpa)]; *i*_cat_ is determined as the current at −0.3 V vs Ag/AgCl. Conditions: 0.1 M phosphate buffer pH 7, Ar atmosphere, 100 mV/s scan rate, 293 K. pH of the solution changed to 6.8 upon addition of 250 mM NaNO_2_.

In measurements in which the concentration of NaNO_2_ is stepwise increased from 100 mM to 1000 mM, the catalytic current increases with nitrite concentration (Figure 3a). To obtain the reaction order in nitrite, the relationship between the nitrite concentration and the observed first-order rate constant (*k*_obs_) should be analyzed. As nitrite reduction is a one-electron process, the electron transfer step is proceeded by one or more rate-limiting chemical steps. For such an EC′-type catalytic mechanism, eq 1 can be applied when the reaction is under kinetic control.^58^ In eq 1, *i*_cat_ is the catalytic current, *i*_p_ is the peak current in the absence of substrate, *n* is the number of electrons transferred, *T* is the temperature (*T* = 293 K), and *v* is the scan rate (*v* = 100 mV/s). From this equation, it follows that *k*_obs_ is proportional to (*i*_cat_/*i*_p_)^2^.
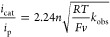
1A plot of (*i*_cat_/*i*_p_)^2^, in which *i*_cat_ is determined as the maximum catalytic current obtained at the peak of the catalytic wave, vs the nitrite concentration shows a linear trend (Figure S5b). At high nitrite concentrations (>500 mM NaNO_2_) the catalytic wave changes from a plateau to a peak-shaped wave, indicative that catalysis becomes a diffusion-controlled process. However, the linear trend found between nitrite concentration and *i*_cat_ over the whole nitrite concentration range indicates that the maximum catalytic current is not affected by these diffusion limitations and eq 1 still holds. For a first-order reaction, the slope of the double-logarithmic plot should be equal to 1, which is the case under these conditions (Figure 3b). Interestingly, repeating the experiments with a lower nitrite concentration range (0.2–100 mM) results in a linear trend (Figure S5a), but the slope of the double-logarithmic plot is 0.77 instead of 1.0 (see Figure S3b). We attribute this change to the disproportionation of NO, which will become a significant competitive reaction for low nitrite concentrations.^11,56,57^ The disproportionation of NO is a redox-neutral process; however, it will indirectly affect the measured *i*_cat_. The *i*_cat_ is inhibited by the formed NO product, as NO will compete with nitrite for binding to copper. At low nitrite concentrations, the relative concentration of produced NO is large and the slope of the double-logarithmic plot will no longer be 1.0. Application of eq 1 to the data in Figure 3a resulted in a *k*_obs_ that increased with increasing [NO_2_^–^] from 98 to 877 s^–1^. Replacing *k*_obs_ in eq 1 by *k*_obs,2_[NO_2_^–^] yields the second-order rate constant (*k*_obs,2_). For *k*_obs,2_, a value of 1.2 × 10^3^ M^–1^s^–1^ was determined from the intercept of the double-logarithmic plot.

In a similar way, the reaction order in catalyst was determined for **Cu(tmpa)** concentrations between 0.2 and 1.0 mM and a nitrite concentration of 250 mM (Figure 3c). The S-shaped catalytic current increases linearly with catalyst concentration (Figure S5d) and becomes diffusion-controlled at high catalyst concentrations. The corresponding double-logarithmic plot has a linear slope of 0.7 (Figure 3d). In line with the nitrite dependence experiments, the linear trend between *i*_cat_ and the catalyst concentration indicates that the maximum catalytic current is not affected by any diffusion limitations. Repeating the measurements with a lower nitrite concentration (20 mM) resulted in a linear dependence of the catalytic current on the catalyst concentration (see Figure S5c). The slope of the double-logarithmic plot is 0.71 for both high and low nitrite concentrations, which is indicative of a side reaction that only affects the catalyst concentration (see Figure S4). Previously, nonelectrochemical studies on **Cu**^**I**^**(tmpa)** have shown that Cu(I) dimers can form in solution both in the absence^59,60^ and presence of NO.^57^ Hence, we assume that similar Cu(I) dimeric species form upon reduction of the catalyst under our electrochemical conditions, resulting in an effective lower catalyst concentration. Taken together, this section shows that there is a linear dependence between the catalytic current and the concentration of nitrite and **Cu(tmpa)**, which indicates that nitrite and **Cu(tmpa)** will most possibly react in a 1:1 ratio.

### Protonation Steps

As mentioned in the introduction, previous research has thus far shed little light on the two protonation steps of the electrocatalytic nitrite reduction at Cu sites. It has been established by Komeda and co-workers that nitrite reduction mediated by **Cu(tmpa)** does not occur at alkaline conditions,^52^ which is evident from the reaction stoichiometry as two protons are required.

**Figure 4:**
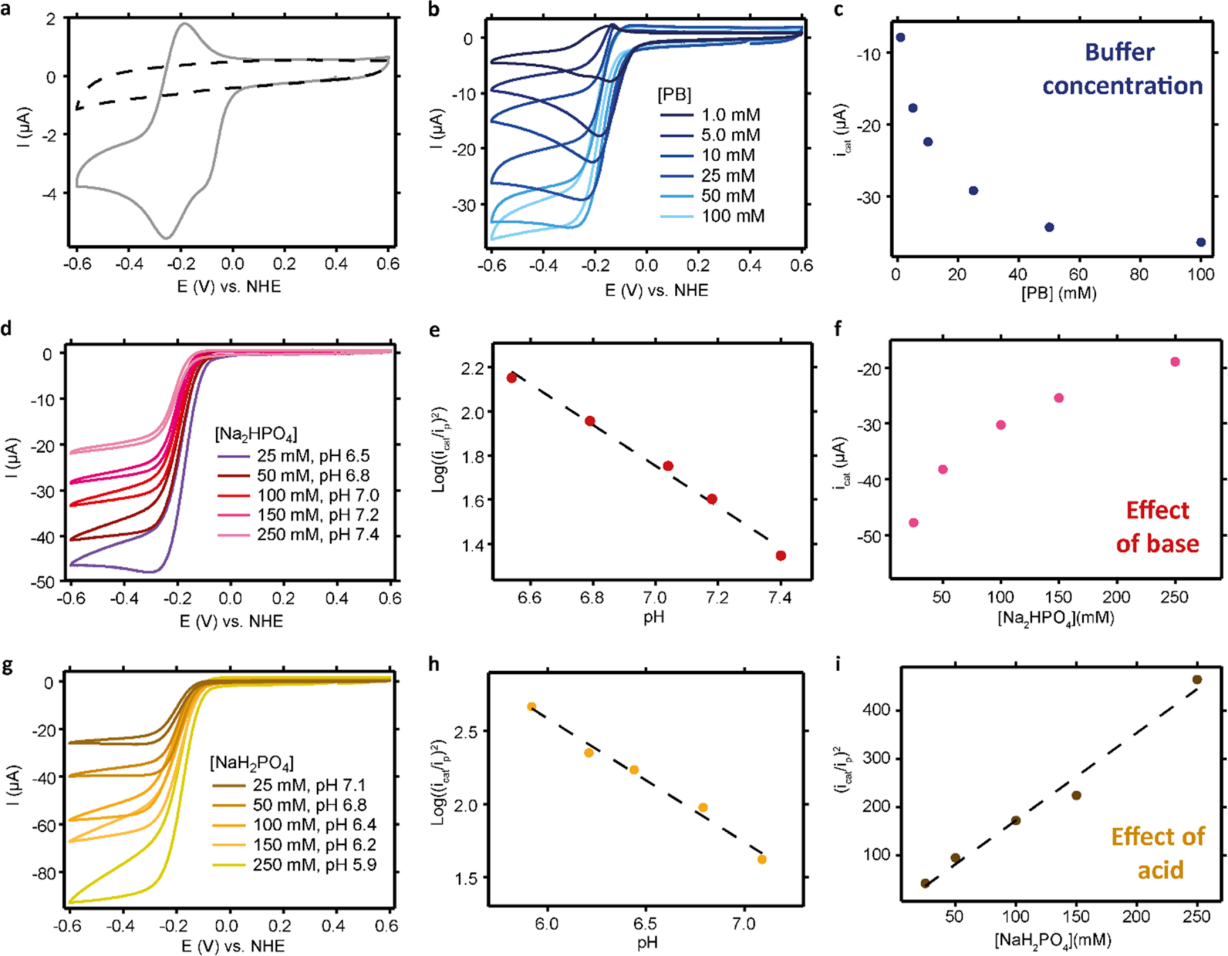
(a) CVs of Cu(tmpa) (gray line) and a bare GC electrode (black dashed line) in 100 mM NaNO_2_ of pH 7 and absence of PB. (b) CVs of Cu(tmpa) in the presence of NaNO_2_ in PB of various concentrations and (c) the corresponding maximum *i*_cat_ measured as a function of [PB]. (d) CVs of Cu(tmpa) in the presence of NaNO_2_ and 50 mM NaH_2_PO_4_, with varying concentrations of Na_2_HPO_4_ and (e) the corresponding logarithmic plot of (*i*_cat_/*i*_p_)^2^ as a function of pH and (f) the corresponding plot of *i*_cat_ as a function of [Na_2_HPO_4_]. (g) CVs of Cu(tmpa) in the presence of NaNO_2_ and 50 mM Na_2_HPO_4_, with varying concentrations of NaH_2_PO_4_ and (h) the corresponding logarithmic plot of (*i*_cat_/*i*_p_)^2^ as a function of pH and (i) the corresponding plot of (*i*_cat_/*i*_p_)^2^ as a function [NaH_2_PO_4_]. In all cases, *i*_cat_ is the current recorded at −0.3 V vs NHE. Conditions: 0.3 mM Cu(tmpa), 20 mM NaNO_2_ (except (a)), Ar atmosphere, 100 mV/s, 293 K.

We hypothesized that the pH and composition of the electrolyte fulfill a crucial role in the reaction mechanism and a set of experiments was carried out to achieve understanding of these processes. The catalytic current disappears when the pH of a 50 mM Na_2_HPO_4_ electrolyte is increased from 6.8 to 8.9, which lies well above the p*K*_a_ of H_2_PO_4_^–^ at 7.2 (see Figure S6). The catalytic activity could not be restored by significantly increasing the concentrations of PB, while maintaining the pH at 11 (Figure S7). Moreover, in the presence of 100 mM of NaNO_2_ at pH 7, but in the absence of buffer, no catalytic current is measured (Figure 4a). These results suggest that H_2_PO_4_^–^ is required for catalysis and hence general acid catalysis is involved in the catalytic mechanism (see Supporting Information Section 5.1 for an elaborate discussion).^61^ This protonation model was further investigated by separately investigating the effect of the buffer concentration ([PB]), base concentration ([Na_2_HPO_4_]), acid concentration ([NaH_2_PO_4_]), and the pH on the catalytic activity.

First, the role of the buffer concentration ([PB]) was studied (Figure 4b). For low buffer concentrations, the catalytic current is peak-shaped, indicating that catalysis is limited by diffusion. In addition, there is a strong dependence of the buffer concentration on the catalytic current when [PB] is low, whereas this effect is minimized when the amount of buffer is increased further. A plot of the catalytic current as a function of buffer concentration supports this explanation (Figure 4c).

The effect of base and the pH was investigated by keeping the concentration of the acid NaH_2_PO_4_ constant at 50 mM, while the concentration of the base Na_2_HPO_4_ was stepwise increased from 25 to 250 mM (Figure 4d). An exponential decrease of the catalytic current as a function of [Na_2_HPO_4_] is observed (Figure 4f), indicating that the base is not involved in catalysis. In addition, the changing base concentration resulted in a pH range of 6.5–7.4 and a linear dependence of log((*i*_cat_/*i*_p_)^2^) on the pH was found (Figure 4e). As the pH of the solution is close to or below the p*K*_a_ of H_2_PO_4_^–^ (p*K*_a_ = 7.2), the pH will affect the concentration of the acid in the buffer (H_2_PO_4_^–^) and hence the observed catalytic current.

Finally, the effect of the acid concentration ([NaH_2_PO_4_]) was studied. The observation that the [H_2_PO_4_^–^] has a direct effect on the catalytic rate illustrates that general acid catalysis is part of the catalytic mechanism. In this experiment, the concentration of Na_2_HPO_4_ was kept constant at 50 mM and the concentration of the acid NaH_2_PO_4_ was varied from 25 to 250 mM, resulting in a pH range from 5.9 to 7.1 (Figure 4g). As these pH values are all below the p*K*_a_ of H_2_PO_4_^–^, the [H_2_PO_4_^–^] is changed directly. Similarly to varying the base concentration, a plot of log((*i*_cat_/*i*_p_)^2^) linearly depends on the pH of the electrolyte when the concentration of NaH_2_PO_4_ is changed (Figure 4h). Moreover, a plot of (*i*_cat_/*i*_p_)^2^ against [NaH_2_PO_4_] shows a linear trend, indicating that H_2_PO_4_^–^ is directly involved in the rate expression (Figure 4i). Simply plotting *i*_cat_ against [NaH_2_PO_4_] provides a linear trend (Figure S8). This contrasts the exponential trend obtained when the [Na_2_HPO_4_] was varied (Figure 4f).

The experiments above provide evidence that general acid catalysis is at play, as the rate of the reaction depends on the H_2_PO_4_^–^ concentration (or buffer) as well as the pH of the electrolyte.^61^ Moreover, this indicates that this general acid-catalyzed proton transfer takes part in the RDS. In line with this, electrochemical studies in D_2_O indicate a significant kinetic isotope effect (KIE) of 2.1 for the nitrite reduction reaction (see Supporting Information Section 6), while no significant changes in the **Cu(tmpa)** redox couple were observed (Figure S9). To our knowledge, no KIE has been published previously for nitrite reduction by a copper-based complex, but such a large KIE can inevitably be attributed to proton transfer to be involved in the RDS.

As the nitrite reduction pathway involves two protons in total, proton inventory experiments were carried out to confirm that only one proton is involved in the RDS. To do so, catalytic currents in different fractions of D_2_O (*n*) were determined in 50 mM phosphate buffer (Figure S10a). This low buffer concentration was chosen as catalysis is independent of buffer concentration above 50 mM (Figure 4b), and it minimizes the proton contribution of the buffer. A plot of (*i*_cat_/*i*_p_)^2^ as a function of the D_2_O fraction results in a linear trend, indicative that the rate-determining step involves a single proton transfer (see Figure S10b). The fractionation factor φ was determined to be 0.44, corresponding to a KIE effect of 2.3 (see Supporting Information Section 6). This value closely matches the value of the KIE effect determined in pure H_2_O and D_2_O.

### Computational Investigations

To study the rate-determining step and uncover further details of the catalytic cycle, density functional theory (DFT) calculations were performed. All DFT calculations were executed using the B3LYP functional including D4 dispersion corrections; a triple zeta basis set with a polarization function (TZP) was used and solvent effects in water were accounted for using the COSMO implicit solvent model (see Supporting Information Section 8).

Before DFT modeling, the possible coordination of nitrite prior to the reduction of **Cu(tmpa)** was investigated. Experiments in organic solvent previously showed that binding of nitrite to **Cu**^**II**^**(tmpa)** results in a distinctive adsorption peak around 410–430 nm.^40^ In the case of an aqueous solution, low nitrite concentrations did not change the ultraviolet–visible (UV–vis) spectrum of **Cu(tmpa)** (Figure S11a). For high nitrite concentrations, the absorption of nitrite itself complicates the interpretation of the UV–vis spectrum (Figure S11b). However, the color of the solution changes in the presence of 250 mM NaNO_2_ from blue to green, which is in line with the formation of a [Cu(tmpa)(NO_2_)]^+^ species (Figure S12).^40^ In addition, the EPR spectrum of **Cu(tmpa)** changes in the presence of high concentrations of nitrite (Figure S13), as shown before by Lehnert and co-workers for similar Cu(II) complexes with tetradentate ligands.^12^ Taken together, we propose that binding of nitrite to **Cu**^**II**^**(tmpa)** is possible, but is in equilibrium with water.

Computed structures of [Cu(tmpa)NO_2_]^+^ show this species indeed is stable in aqueous solution. Analysis of the nitrite binding mode in [Cu(tmpa)NO_2_]^+^ reveals that the energy difference between the N- and O-bound complexes is less than 0.3 kcal/mol (Table S1 entries 1–3, and Figures S14–16), suggesting that both binding modes can coexist. Modeling of the bidentate η_2_-O,O-bonded nitrite structure resulted in a species that is around 2.0 kcal/mol higher in energy than both monodentate bound nitrite complexes. In addition, one of the Cu–O bonds is 2.9 Å, which indicates that bidentate binding of nitrite is not favored (Figure S15). In agreement with our calculations, crystal structures of [Cu(tmpa)NO_2_]^+^ with both the κ-N and κ-O nitrite binding modes were reported previously.^37,40,52^ The monodentate binding of nitrite to **Cu(tmpa)** deviates from other nitrite-bound Cu(II) complexes, as the η_2_-O,O binding mode is observed in the majority of reported crystal structures with ligands other than tmpa.^22,26−29,31−33,35,36,41^ Karlin and co-workers attributed the preference of **Cu(tmpa)** for the monodentate binding of nitrite to the tetradentate nature of the tmpa ligand.^41^

Calculated structures of the reduced [Cu(tmpa)NO_2_] species show that binding of nitrite to Cu(I) by its nitrogen atom is energetically favored over the O-bound structure by 2.1 kcal/mol (Table S1 entries 4–6 and Figures S17 and S18). The calculated structure of [Cu(tmpa)NO_2_] with η_2_-O,O-bonded nitrite has one elongated Cu–O bond of 3.0 Å, indicative that bidentate binding is not favored (Figure S19). The binding mode of nitrite to **Cu**^**I**^**(tmpa)** has not been reported before; nevertheless, our results are consistent with other N-bound mononuclear Cu(I) nitrito species in the literature.^19,21−23,26−30^ Besides, only N-bound nitrosyl copper complexes have been reported thus far outside an enzymatic environment,^19,27,42,57^ suggesting that dehydration takes place from the N-bound nitrite.

After reduction of the complex and binding of nitrite, the subsequent protonation steps were investigated. It is unlikely that NO_2_^–^ will be protonated to HNO_2_ prior to binding to **Cu**^**I**^**(tmpa)**. The p*K*_a_ of HNO_2_ is 3.15, and the concentration of HNO_2_ will only be 2.8 μM when 20 mM NaNO_2_ is added to PB of pH 7. In line with the above findings, geometry optimization calculations indicate that protonation of the N-bound nitrite complex [Cu(tmpa)NO_2_] leading to [Cu(tmpa)HNO_2_]^+^ is energetically favored over protonation of the O-bound analogue by 3.1 kcal/mol (Table S1 entries 7–8, and Figures S20 and S21). Besides, our calculations indicate that the separate protonation of both O-atoms on NO_2_^–^ is an uphill reaction of 39.2 kcal/mol compared to the double protonation of only one O atom (Table S1 entries 9–10, and Figures S22 and S23).

**Figure 5:**
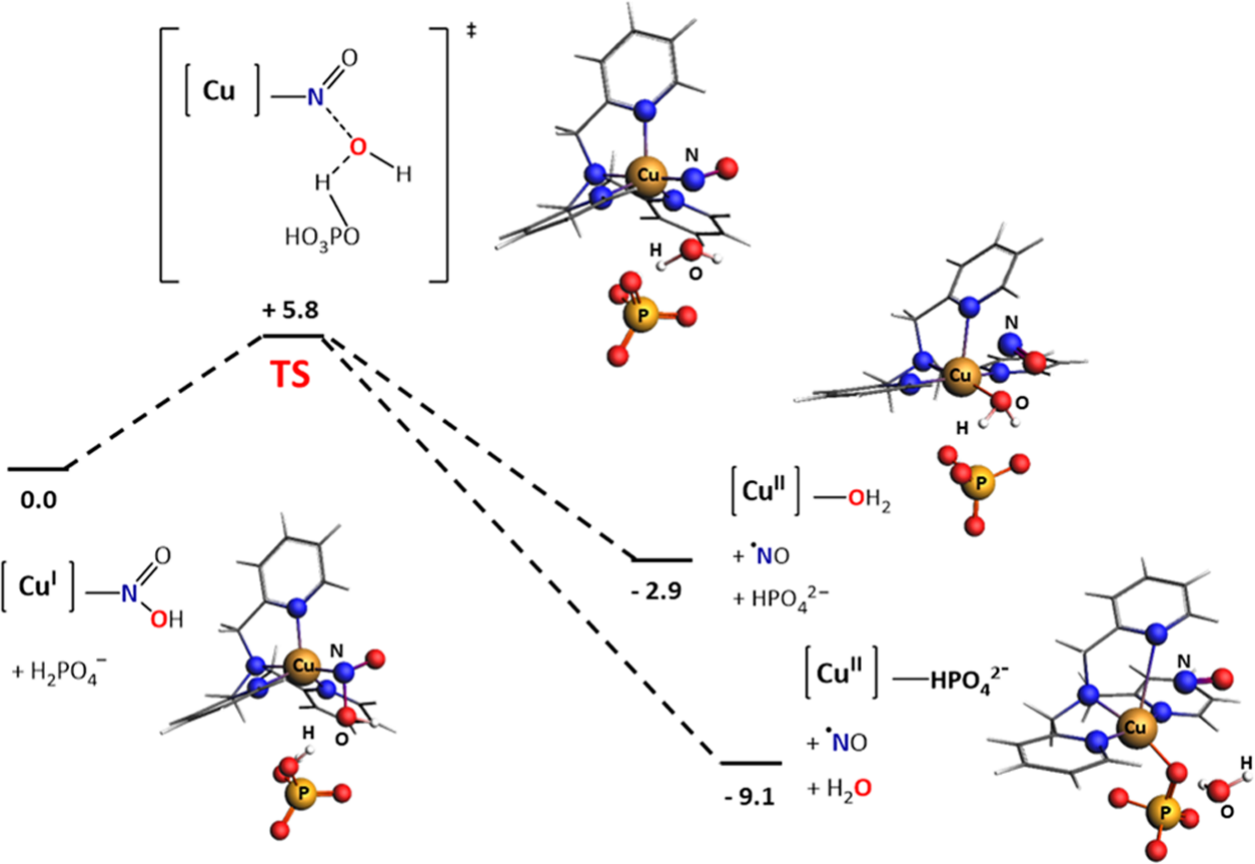
Proposed pathway of the protonation of [Cu(tmpa)HNO_2_]^+^ by H_2_PO_4_^–^ to form [Cu(tmpa)H_2_O]^2+^ or [Cu(tmpa)HPO_4_] via the found transition state (TS) of [Cu(tmpa)NO]^2+^. Gibbs free energies are given in kcal/mol at 298 K and 1 atm. Color scheme: Cu, brown; O, red; N, blue; P, yellow; H, white.

The electrochemical measurements provided evidence that the protonation steps in nitrite reduction can be separated in a rate-limiting, general acid-catalyzed protonation, and a separate protonation by the solvent. It is assumed that the second protonation resulting in N–O bond breaking, is the RDS. The energy profile of the RDS, in which a proton is delivered by H_2_PO_4_^–^, was therefore modeled by DFT (Figure 5). A scan of H_2_PO_4_^–^ approaching [Cu(tmpa)(HNO_2_)]^+^ resulted in the identification of a transition state structure (Figure S24). In this transition state a proton from the incoming H_2_PO_4_^–^ species is transferred to the O atom of HNO_2_ with H–O and O–N distances of 1.23 and 2.13 Å, respectively. The energy barrier of the proton transfer was determined to be only 5.8 kcal/mol (Table S1 entries 11–12, and Figures S24 and S25). Additionally, our calculations show that the loss of the water molecule and protonation take place in a concerted manner. As a control, attempts to find a transition state for the first protonation of NO_2_^–^ by H_2_PO_4_^–^ did not succeed, indicating that the first proton is delivered by the solvent in a barrierless step.

The above calculations were carried out in a spin-restricted approach, as the Cu(I) species are expected to be diamagnetic. However, we hypothesize that in the product state, the formed NO will leave the copper site, resulting in a d^9^ Cu(II) complex and a free NO^•^ molecule. In a geometry optimization of the formed [Cu(tmpa)NO]^2+^ complex for the triplet state, the NO ligand is readily replaced by a water molecule or HPO_4_^2–^, resulting in two local minima that are 2.9 and 9.1 kcal/mol lower in energy than the reactants, respectively (Table S1 entries 12, 14, and 15, Figures S25, S27, and S28). This indicates that NO will dissociate the copper site upon its formation and reduction of [Cu(tmpa)H_2_O]^2+^ or [Cu(tmpa)HPO_4_] can take place again. The nitrosyl complex, [Cu(tmpa)NO]^2+^, that is formed as an intermediate at the end of the catalytic reaction is relevant for the further conversion of NO to N_2_O.^11,56,57^ However, elucidation of the electronic structure of the Cu(II)–NO species is challenging due to the noninnocent nature of the NO ligand and therefore was not further investigated. In this light, previously only one crystal structure of a Cu(II)–NO complex has been published by Hayton and co-workers^42^ as isolation of Cu(II)–NO species is in general challenging.^19^ In addition, studies on enzymatic copper nitrosyl species, especially Cu(I)–NO, have been shown to be complicated due to the different binding modes of NO to copper.^2,46,62,63^

### Nitrite Reduction Mechanism

**Figure 6:**
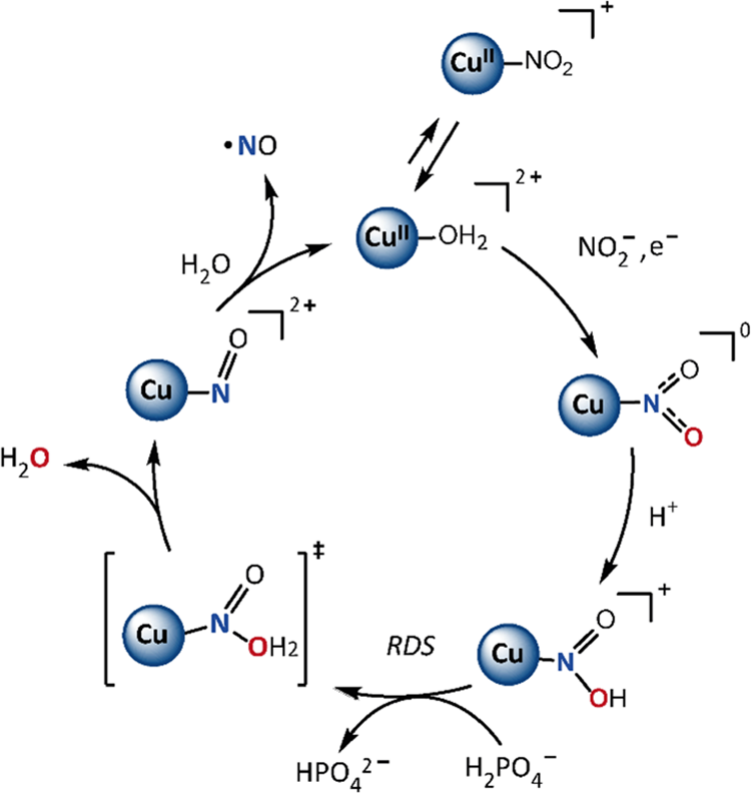
Schematic overview of the proposed catalytic cycle of nitrite reduction catalyzed by Cu(tmpa) in aqueous PB of pH 7. The tmpa ligand is omitted for clarity.

The proposed mechanism of electrochemical nitrite reduction to nitric oxide catalyzed by **Cu(tmpa)** in PB pH 7 is shown in Figure 6. Starting from the oxidized catalyst, formation of the [Cu(tmpa)NO_2_]^2+^ species is observed only at high nitrite concentrations, which indicates that binding of nitrite to Cu^II^(tmpa) is in equilibrium with water. In the majority of electrochemistry experiments low nitrite concentrations are used and the largest part of the bound water molecules will not be replaced by nitrite until reduction of Cu(II) to Cu(I). Investigation of the dependence of the catalytic current on the nitrite and catalyst concentration illustrated that **Cu(tmpa)** will react with nitrite in a 1:1 ratio. Additionally, investigation of the effect of the catalyst concentration indicates that Cu(I) dimeric species might form during catalysis. In previous studies by our group on the reduction of oxygen and hydrogen peroxide by **Cu(tmpa)** in the same buffer, formation of any such dimeric species was not observed.^64,65^ We attribute this discrepancy to the large differences between the observed rate constants for the reduction of nitrite (9.0 × 10^2^ s^–1^ from Figure 3a), oxygen (1.8 × 10^6^ s^–1^), and hydrogen peroxide (2.1 × 10^5^ s^–1^), allowing the Cu(I) species more time to form a dimer only during reduction of nitrite.

From DFT calculations it is evident that for [Cu(tmpa)NO_2_] the N-bound nitrite species is energetically favored over the O-bound species. In addition, DFT calculations indicate that protonation of the N-bound nitrite is favored over protonation of the O-bound analogue, validating that the mechanism of nitrite reduction takes place via the N-bound pathway. Regarding the protonation steps, proton inventory experiments and a first-order dependence in [H_2_PO_4_^–^] point to an RDS in which only one proton is involved. Based on this, the first protonation is a pre-equilibrium protonation by the solvent, while the second protonation is the RDS that results in breaking of the N–O bond. Electrochemical measurements provided evidence that this RDS is generally acid-catalyzed. In a PB solution of pH 7, it is expected the proton will originate from H_2_PO_4_^–^, while at lower pH values H_3_O^+^ and HNO_2_ are possible proton sources too. Modeling of the RDS by DFT calculations indicates that the transfer of a proton from H_2_PO_4_^–^ to [Cu(tmpa)HNO_2_]^+^ has an energy barrier of 5.8 kcal/mol and that protonation and release of H_2_O take place in a concerted manner. The calculated energy barrier of this reaction is small, especially for a relatively low *k*_obs_. A reason for this small barrier might be that the calculated energy of the reactant state is too high since the stabilizing hydrogen-bonding network cannot be accurately modeled by the implicit solvent model. DFT calculations indicate that the NO ligand will be replaced by a molecule of water or HPO_4_^2–^, from which electron transfer can take place again.

## Conclusions and Outlook

This work presents for the first time a detailed description of the electrocatalytic nitrite reduction mechanism by a bio-inspired CuNiRs catalyst in neutral, aqueous solution. We show that for **Cu(tmpa)** the electron and proton transfer steps take place in a stepwise manner during nitrite reduction. Electron transfer is followed by a pre-equilibrium protonation reaction of Cu-bound NO_2_^–^. Next, a general acid-catalyzed proton transfer takes place during the RDS. Modeling of this RDS by DFT supports this mechanism and shows that NO is released from the Cu site at the end of the catalytic cycle. Most importantly, at neutral pH, the proton in the RDS originates from the acid in phosphate buffer, NaH_2_PO_4_, which is thus part of the rate expression.

Though in recent years research has focused on the investigation of the protonation steps in CuNiRs,^53,63,66−69^ the protonation steps in bio-inspired CuNiRs are regularly overlooked. This is surprising as to our knowledge no studies on homogeneous copper catalysts exist that can perform nitrite reduction in neutral solutions in the absence of a buffer, suggesting that general acid catalysis is a uniform requirement for nitrite reduction at mononuclear copper sites. In a recent paper by Lehnert and co-workers, the authors state that additional experiments to explore the pH dependence of nitrite reduction by **Cu(tmpa)** and similar complexes were not conducted as the rate of NO_2_^–^ decomposition increases at low pH.^12^ Statements of this kind point out the need to optimize catalytic activities in neutral, aqueous solution, wherefore it is essential to completely understand how the catalytic activity is established.

Our results suggest that the catalytic activity of similar reported copper complexes is greatly dependent on the reaction conditions. Consequently, a fair comparison of the activity between different catalysts thus requires that the pH and acid concentration are considered. Thus far, such comparisons are not being made.^48^ This not only complicates any meaningful comparison of catalytic activity, but it is also disadvantageous for the development of new catalytic systems.

We therefore urge future research on bio-inspired CuNiRs and related catalytic systems to carefully address the role of the electrolyte and use these measurements as a guideline.

In addition, the insights presented herein are valuable for applications that use copper-based catalysts and require neutral, aqueous solutions, like wastewater treatment or biomedical applications. Considering the growing urge to selectively and electrochemically convert nitrogen-containing compounds like nitrite to restore the global nitrogen cycle,^4,5^ this work contributes to improving the electrochemical analysis of bio-inspired CuNiRs catalysts, which is inevitable for their further development.

## Supporting Information Available

Experimental information, electrochemical experiments, UV–vis spectra, EPR spectra, calculated structures, energies, and Cartesian coordinates (PDF)
