# Supplementary material for: Elucidation of the Electrocatalytic Nitrite Reduction Mechanism by Bio-Inspired Copper Complexes
Source: ACS Catal. 2023 Jul 18;13(15):10094–103. doi: 10.1021/acscatal.3c01989 (PMC10407843; doi:10.1021/acscatal.3c01989)
Supplement: Supplementary file 1 — cs3c01989_si_001.pdf [file cs3c01989_si_001.pdf]

Supporting Information

Elucidation of the Electrocatalytic Nitrite Reduction Mechanism by Biomimetic Copper Complexes

Phebe H. van Langevelde[a], Silène Engbers[a], Francesco Buda[a], and Dennis G. H. Hetterscheid[a*]

[a]Leiden Institute of Chemistry, Leiden University, 2300 RA, Leiden (The Netherlands)

*Corresponding author: Dennis G. H. Hetterscheid, email: d.g.h.hetterscheid@chem.leidenuniv.nl.

Content

1. Experimental ... 3
1.1 General ... 3
1.2 Electrochemistry measurements ... 3
1.2.1 Proton inventory experiments ... 4
2. Rotating Disk Electrode measurements ... 5
3. Homogeneity of Cu(tmpa) during electrochemistry ... 6
4. Reaction order in nitrite and catalyst concentration ... 7
5. Nature of the protonation steps ... 9
5.1 Explanation of general and specific acid catalysis ... 9
5.2 The effect of buffer on the catalytic activity ... 9
6. Proton inventory experiments ... 10
7. UV-vis study of Cu(tmpa) solutions in the presence of nitrite ... 13
8. EPR spectra of Cu(tmpa) solutions in the presence of nitrite ... 14
9. DFT calculations ... 15
9.1 Binding of nitrite to Cu(II)tmpa ... 16
9.2 Binding of nitrite to Cu(I)tmpa ... 18
9.3 Binding of HNO_2 to Cu(I)tmpa ... 20
9.4 Second protonation step ... 21
9.5 Energy profile of the RDS ... 22
9.6 XYZ coordinates of optimized structures ... 26
References ... 42

1. Experimental

1.1 General

All chemicals and solvents were purchased from commercial chemical suppliers without further purification. All electrolyte solutions for electrochemistry were prepared using Mili-Q grade water and high purity salts, $NaNO_2$ (99.999% trace metals basis, Sigma Aldrich), NaH_2PO_4 (99.99% Suprapur, Merck), Na_2HPO_4 (≥99.999% TraceSELECT, Honeywell Fluka). D_2O for the proton inventory experiments was obtained from Eurisotop (99.9 % D). The Ar gas used in electrochemical experiments was supplied by Linde. The pH of all electrolyte solutions was measured using a HI 4222 pH meter from Hanna Instruments. UV-vis measurements were conducted on a Agilent Varian Cary 50 spectrophotometer. EPR spectra were recorded on a Bruker EMXPlus X-band spectrometer. $[Cu(tmpa)MeCN]^{2+}$ (tmpa = tris(2pyridylmethyl)amine) was synthesized as previously reported.[1]

1.2 Electrochemistry measurements

All electrochemical experiments were carried out in custom-build glass cells using a three-electrode setup. In all measurements the working electrode was made from glassy carbon (A = 0.71 cm^2, Metrohm), encapsulated in PEEK. The working electrode was polished on a Struers LaboPol-30 polishing machine in two steps. First the GC surface was cleaned with diamond polish (1.0 µm, DiaPro, Struers) on polishing cloth (Dur-type) for 1 minute. Next, the electrode was polished on a different polishing cloth from the same material with silica suspension (OP-S Non-dry, Struers) for 2 more minutes. After this, the electrode was sonicated in Mili-Q for 15 minutes. In all CV measurements the counter electrode was made of a Au wire, which was flame annealed and rinsed with Mili-Q at the start of the day. The reference electrode in all experiments was a double junction Ag/AgCl electrode (3M KCl, Metrohm) of which the equilibrium potential was regularly measured versus a reversible hydrogen electrode (RHE). All glassware for electrochemistry measurements was regularly cleaned by overnight soaking in an aqueous solution of $KMnO_4$ (1 g/l) and H_2SO_4 (0.5 M), after which it was further cleaned in a dilute solution of H_2O_2 and H_2SO_4 and boiled three times for 30 minutes in Mili-Q water. Prior to every measurement, all glassware was boiled in Mili-Q for 30 minutes more. All electrochemical measurements were conducted on PGSTAT 12, 204 or 128N potentiostats from Autolab, using NOVA software. Prior to recording a CV measurement in presence of

catalyst, three CV scans were always recorded in absence of catalyst in a blank electrolyte solution to ensure the cleanliness of the working electrode. In addition, the electrode surface of the working electrode was polished every time after recording CVs in presence of catalyst.

1.2.1 Proton inventory experiments

Prior to all proton inventory experiments, all glassware was placed in an oven at 160 °C overnight. In addition, [Cu(tmpa)(CH_3CN)]OTf_2, $NaNO_2$, Na_2HPO_4, and NaH_2PO_4 were dried in a vacuum oven for one hour before preparing the electrolyte solutions. A 50 mM phosphate buffer solution was prepared in both D_2O and H_2O, and the pH of both solutions was measured using a H_2O-calibrated pH meter. Using **Equation 1**,[2] the measured pH of the D_2O solution (7.1) was converted, obtaining a pH_{D2O} of 7.0. The electrodes were prepared as described above, except that the working electrode was sonicated in D_2O after polishing, and the counter electrode and reference electrode were rinsed with D_2O prior to the measurement. As experiments are very sensitive for the D_2O fraction, the surface of the working electrode was saturated by the correct D_2O fraction by measuring 10 CV cycles in a blank solution in absence of catalyst prior to recording the catalytic activity. In addition, prior to every experiment, the working electrode was soaked in the catalyst solution for a few minutes before recording the CV scans.

$$pH = 0.929pH_{D_2O} + 0.41 \qquad (\mathbf{1})$$

2. Rotating Disk Electrode measurements

All rotating disk electrode (RDE) experiments were carried out with an Autolab PGSTAT 12 potentiostat and a MSR rotator from Pine Instruments. All RDE measurements were carried out using a custom-made three-electrode electrochemical cell with a volume of more than 40 ml, in which the counter electrode compartment is separated from the bulk solution by a glass frit. The glassware was cleaned in a similar manner as described for stationary electrochemistry experiments. A Pine Instruments ChangeDisk RDE electrode (E5TQPK) with a GC disk of 5 mm diameter working electrode and a gold counter electrode were used. The working electrode and counter electrode were cleaned and prepared as described above. A RHE reference electrode, made from a Pt mesh and filled with a bubbled H_2 buffer solution, was connected to the main cell compartment via Luggin capillary. The electrolyte solution was bubbled with Ar gas for at least 25 minutes prior to the experiment. Also during the experiment Ar gas was bubbled through the electrolyte to keep oxygen out of the solution.

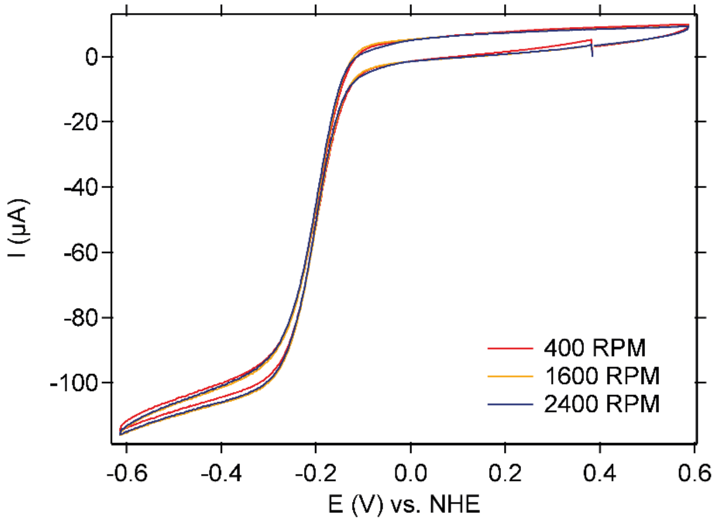

Figure S1 RDE CVs of **Cu(tmpa)** recorded in the presence of 20 mM $NaNO_2$ at different rotation rates. Conditions: 0.1 M PB pH 7, 0.3 mM Cu(tmpa), 20 mM $NaNO_2$, Ar atmosphere, 293 K, 50 mV/s scan rate.

3. Homogeneity of Cu(tmpa) during electrochemistry

The homogeneity of the catalyst during nitrite reduction catalysis was investigated in CV experiments (**Figure S2**). In these measurements a CV of **Cu(tmpa)** was recorded in presence of high concentrations of nitrite ([$NaNO_2$] = 250 mM). Thereafter the GC electrode was thoroughly rinsed with Mili-Q water and the same GC electrode was used to record a CV in a solution of 250 mM $NaNO_2$ in absence of catalyst. From these measurements it is evident that the CV of the GC electrode in absence of catalyst does not change. This shows that no catalyst sticks to the surface of the electrode, nor do any catalytically active heterogeneous deposits form during catalysis.

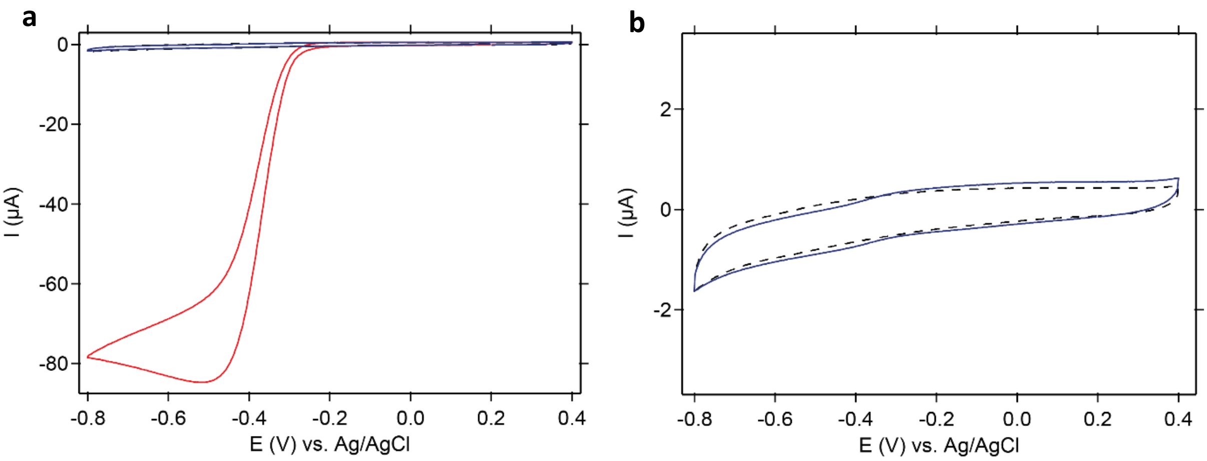

Figure S2 a) CV of the catalytic activity of Cu(tmpa) recorded in the presence of 250 mM $NaNO_2$ (red line), compared to the activity of the GC electrode before (black dashed line) and after recording 6 catalytic CV scans in the presence of 250 mM $NaNO_2$ (blue line). **b)** Zoom-in of the blue and black dashed lines in **a)**. Conditions: 0.1 M PB pH 7, 0.3 mM Cu(tmpa), 250 mM $NaNO_2$, Ar atmosphere, 293 K, 100 mV/s scan rate.

4. Reaction order in nitrite and catalyst concentration

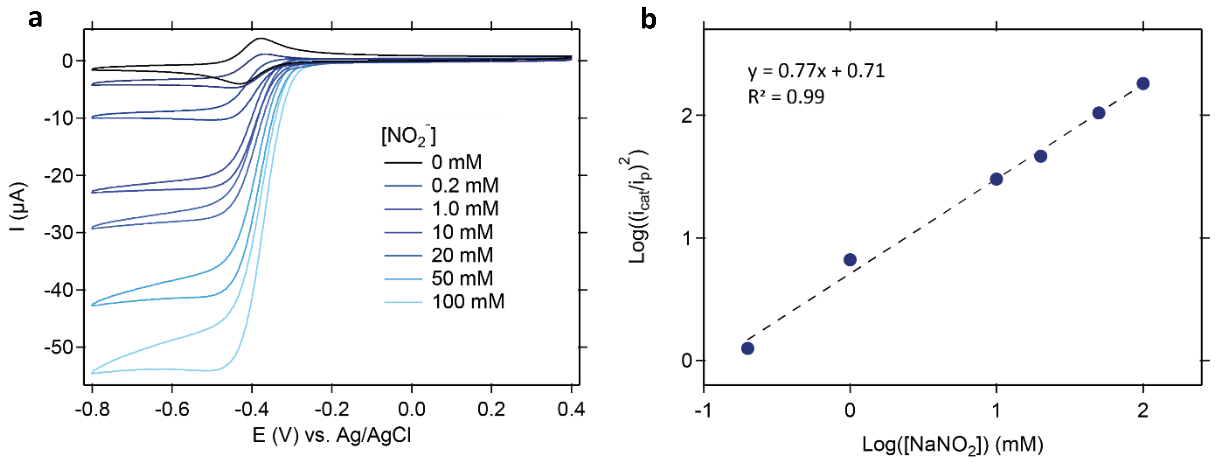

Figure S3 a) CVs of **Cu(tmpa)** in the presence of 0 – 100 mM NO_2^- recorded under Ar atmosphere. **b)** Corresponding logarithmic plot of $(i_{cat}/i_p)^2$ as a function of $[NaNO_2]$, i_{cat} is determined as the current at -0.5 V vs. Ag/AgCl. Reaction conditions: 0.3 mM **Cu(tmpa)**, 0.1 M phosphate buffer pH 7, Ar atmosphere, 293 K, 100 mV/s scan rate.

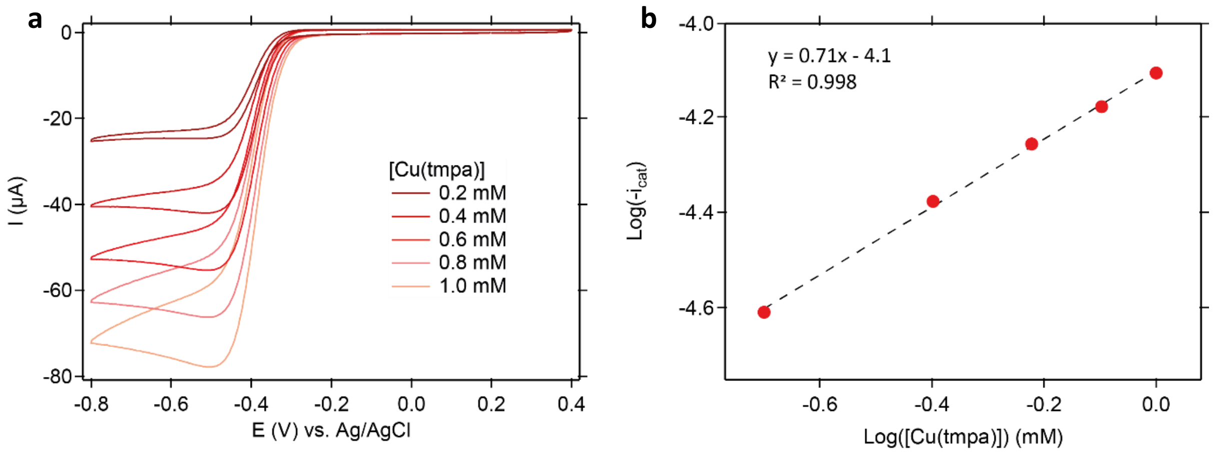

Figure S4 a) CVs of different **Cu(tmpa)** concentrations (0.2 – 1.0 mM) in the presence 20 mM NO_2^- recorded under Ar atmosphere. **b)** Corresponding logarithmic plot of $-i_{cat}$ as a function of [Cu(tmpa)], i_{cat} is determined as the current at -0.5 V vs. Ag/AgCl. Reaction conditions: 250 mM $NaNO_2$, 0.1 M phosphate buffer pH 7 (pH changed to 6.8 upon addition of 250 mM $NaNO_2$), Ar atmosphere, 293 K, 100 mV/s scan rate.

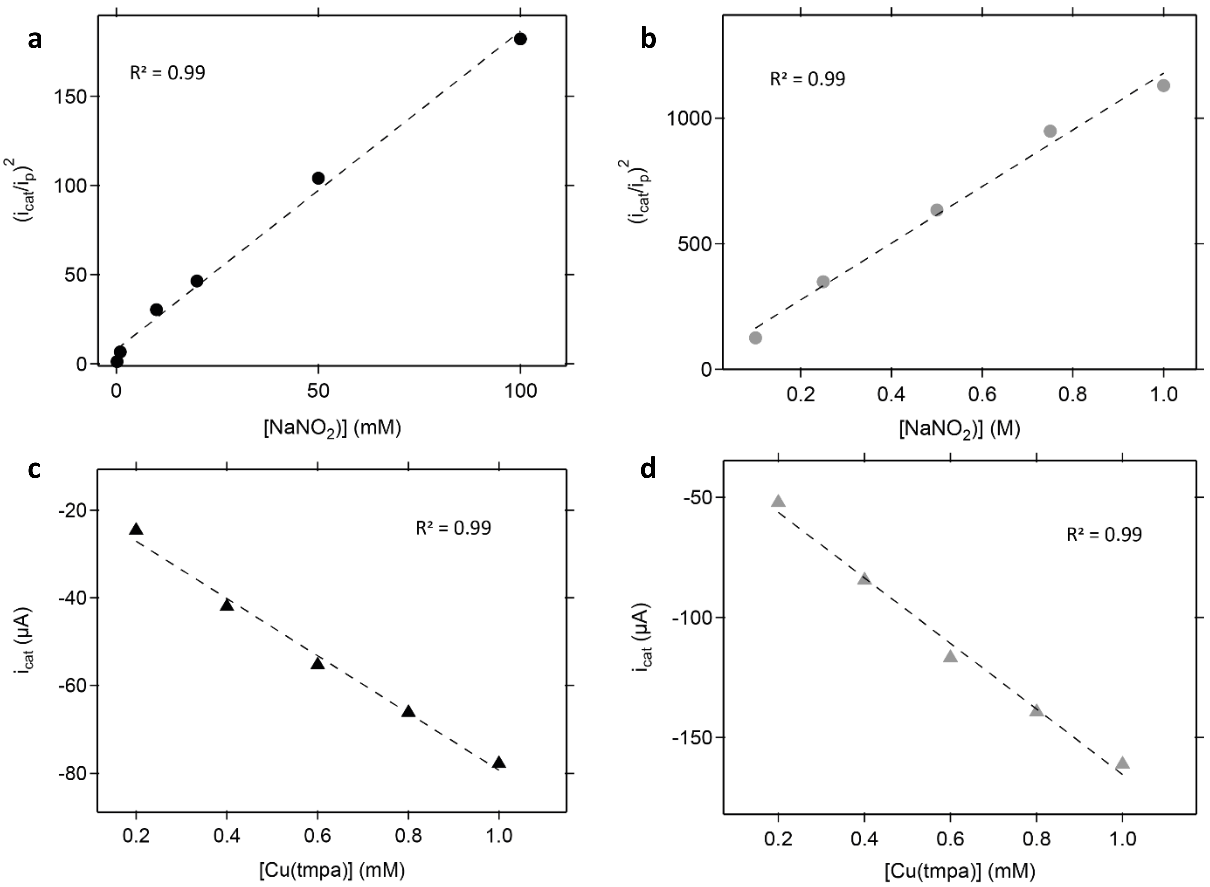

Figure S5 Figure S5 Plots of $(i_{cat}/i_p)^2$ as a function of [$NaNO_2$] between a) 0 - 0.1 M or b) 0.1 - 1.0 M determined for 0.3 mM Cu(tmpa). And plots of i_{cat} as a function of [Cu(tmpa)] between 0.2 - 1.0 mM determined in presence of c) 20 mM or d) 250 mM $NaNO_2$. Conditions: 0.1 M phosphate buffer pH 7, Ar atmosphere, 100 mV/s scan rate, 293 K.

5. Nature of the protonation steps

5.1 Explanation of general and specific acid catalysis

In general acid catalysis, proton transfer occurs in the rate determining step (RDS) and the rate for $A + HB \rightarrow HA^{+} + B^{-}$ can be expressed as $r = k_{obs}[A][HB]$.[3] Taking into account the potential acids present in the reaction mixture (aqueous PB) this would lead to $r \sim k_1[H_3O^{+}] + k_2[H_3PO_4] + k_3[HNO_2] + k_4[H_2PO_4{}^{-}] + k_5[HPO_4{}^{2-}] + +k_6[H_2O]$. Here it is important to realize that the concentrations of the acids with a low pK_a are too low to contribute significantly, and the acids with a high pK_a are probably insufficiently reactive. This often leads to a catalytic reaction that is most dependent on the acidic component of the phosphate buffer, and typically this effect is most prominent at neutral pH. In the other extreme case, the protonation reaction occurs prior to the rate determining step, and a pre-equilibrium approximation can be applied in the rate equation. This leads to a situation wherein the rate law depends on the acid/base equilibrium resulting in $r \sim \frac{[HB][A]}{[B^{-}]}$. In this situation the catalytic rate will be independent of the buffer concentration, given that at the buffering pH the ratio $\frac{[BH]}{[B^{-}]}$ remains identical. Hence by studying the dependence of the catalytic reaction on the buffer concentration one can learn if general acid catalysis is at play.

5.2 The effect of buffer on the catalytic activity

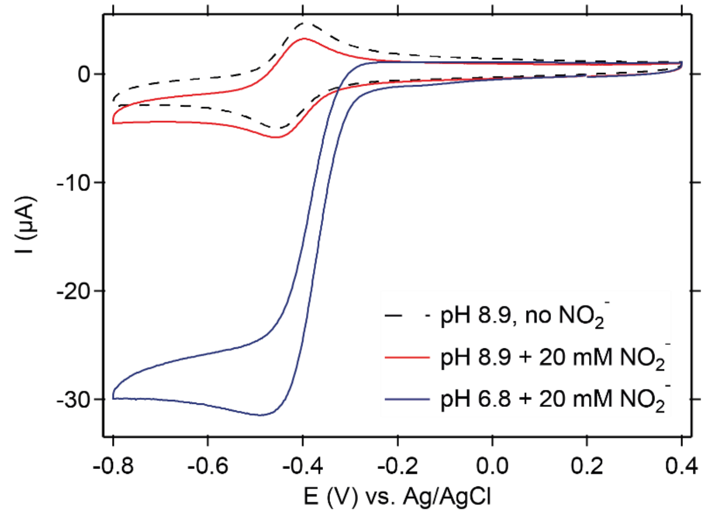

Figure S6 CV measurements of the catalytic activity of **Cu(tmpa)** in absence (black dashed line) and in presence of 20 mM $NaNO_2$ at pH of 8.9 (red line) and at pH 6.8 (blue line). Conditions: 0.3 mM Cu(tmpa), 50 mM Na_2HPO_4, Ar atmosphere, 293 K, 100 mV/s scan rate.

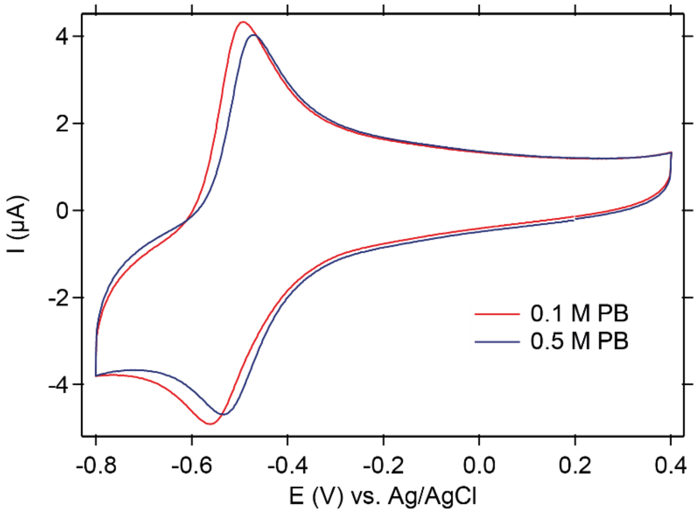

Figure S7 CV measurements of **Cu(tmpa)** in the presence of 20 mM NaNO2 in 0.1 M PB of pH 11.3 (red trace) and in 0.5 M PB of pH 11.0 (blue trace). Conditions: 0.3 mM Cu(tmpa), Ar atmosphere, 293 K, 100 mV/s scan rate.

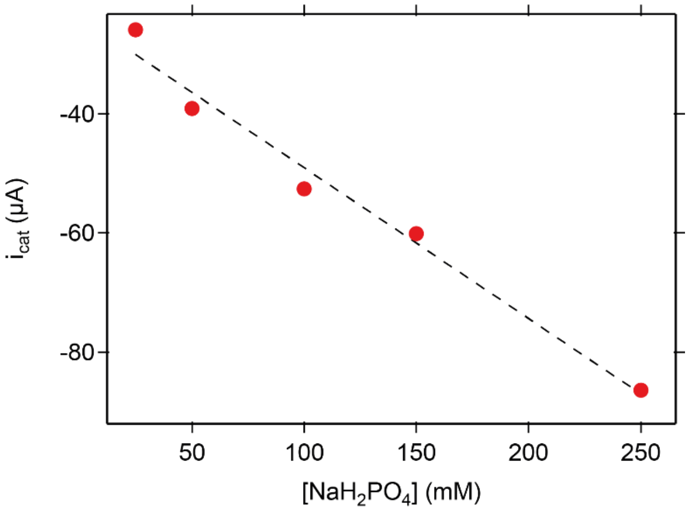

Figure S8 Plot of -i_{cat} as a function of [NaH_2PO_4] in the presence of 50 mM Na_2HPO_4. i_{cat} is determined as the current at -0.5 V vs. Ag/AgCl. Reaction conditions: 0.3 mM **Cu(tmpa)**, 20 mM $NaNO_2$, Ar atmosphere, 100 mV/s scan rate, 293 K.

6. Proton inventory experiments

Proton inventory experiments were carried out by recording CVs of **Cu(tmpa)** in solutions with varying fractions of D_2O in the absence of substrate (See **Figure S9**) and in the presence of nitrite (See **Figure S10a**). The basis of a proton inventory experiment is that each proton in the rate-determining step has its own fractionation factor (φ), which describes the extent of protonation at the transition state and is equal to k_D/k_H, the inverse of a kinetic isotope effect. The overall value of k_D/k_H is a multiplication of the individual φ values of each proton involved. By varying the D_2O fraction (n) the φ values can be deconvoluted using **Equation 2**. A plot of k_n/k_H against n will only lead to a linear relationship if one proton is involved in the rate determining step, and a quadratic one if two protons are involved that have a different fractionation factor.[4]

$$\frac{K_{obs}^{n}}{K_{obs}^{H}} = (1 - n + \varphi_1)(1 - n + \varphi_2) \ldots \quad (\mathbf{2})$$

In order to determine the fractionation factor, a plot of $(i_{cat}/i_p)^2{}_n$ / $(i_{cat}/i_p)^2{}_H$ was prepared, in which n represents the fraction of D_2O and H represents the measurements in pure H_2O (See **Figure S10b**). The value of i_p in this plot was determined as the average current of all anodic peaks in **Figure S9**, and i_{cat} was determined for every D_2O fraction as the average catalytic current recorded at -0.5 V vs. Ag/AgCl in four separate measurements (**Figure S10a**). The standard value of the kinetic isotope effect (KIE) was determined according to **Equation 2**, using $(i_{cat}/i_p)^2{}_D$ in pure D_2O and $(i_{cat}/i_p)^2{}_H$ in pure H_2O, which are proportional to $k_{obs,D}$ and $k_{obs,H}$, respectively.

$$KIE = \frac{k_{obs,H}}{k_{obs,D}} \propto \qquad (\mathbf{2})$$

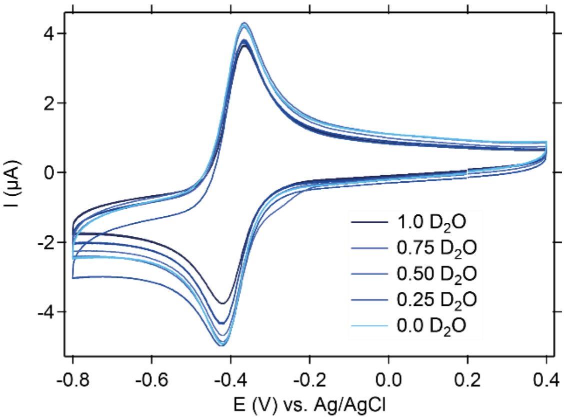

Figure S9 CV measurements of the Cu(tmpa) redox couple measured for varying D_2O fractions in H_2O between 0.0 and 1.0. Conditions: 0.3 mM Cu(tmpa), 0.05 M PB, Ar atmosphere, 100 mV/s scan rate, 293 K.

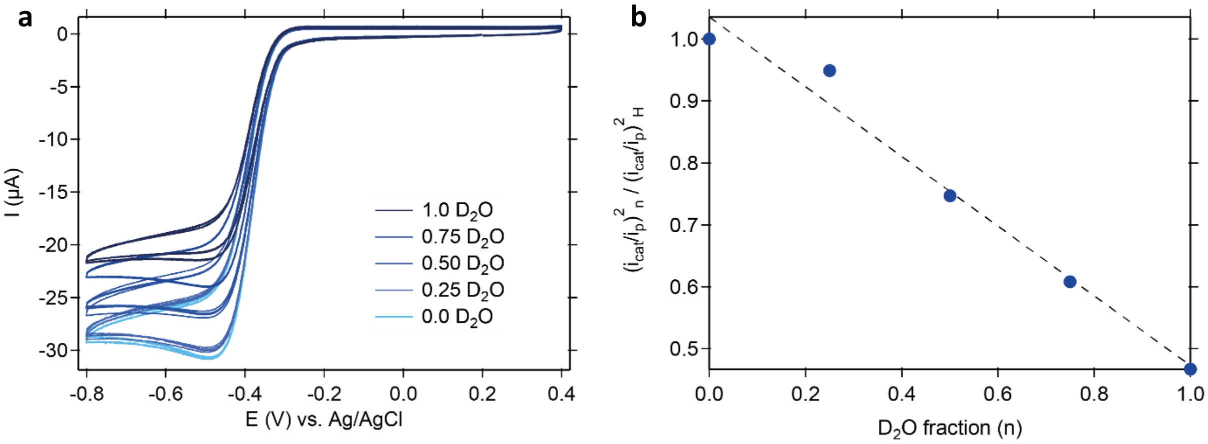

Figure S10 a) CV measurements of 3 scans of **Cu(tmpa)** in the presence of 20 mM $NaNO_2$ with D_2O fractions between 0.0 and 1.0. b) Corresponding plot of $(i_{cat}/i_p)^2$ as a function of the D_2O fraction. Conditions: 0.3 mM Cu(tmpa), 0.05 M PB pH 7, Ar atmosphere, 100 mV/s scan rate, 293 K. i_{cat} was determined as the average maximum catalytic current from 4 separate measurements.

7. UV-vis study of Cu(tmpa) solutions in the presence of nitrite

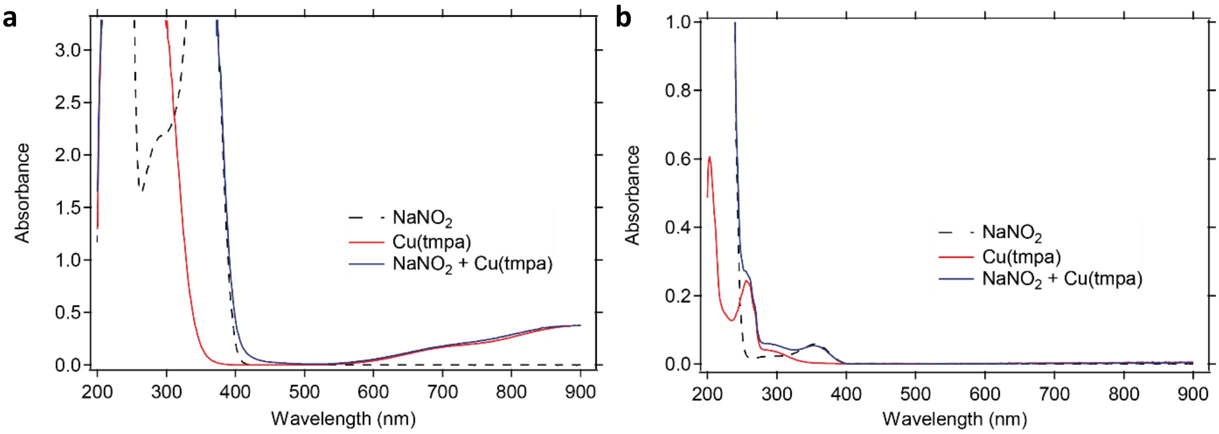

Figure S11 UV-vis spectrum of **a)** 250 mM $NaNO_2$ (black dashed line), 2.0 mM Cu(tmpa) (red line), and 250 mM $NaNO_2$ and 0.2 mM Cu(tmpa) together (blue line) and **b)** Same measurements as in figure a), but solution 100x diluted. Conditions: 250 mM $NaNO_2$, 0.2 mM Cu(tmpa), 0.1 M PB pH 7 (changed to pH 6.8 upon addition of 250 mM $NaNO_2$).

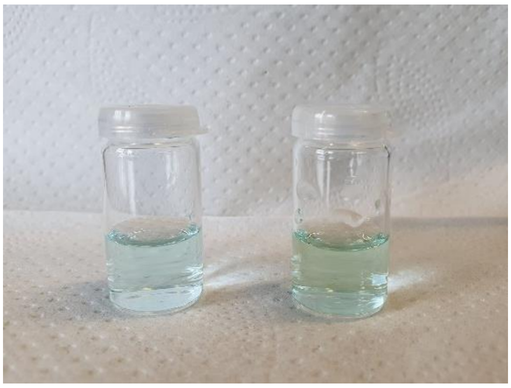

Figure S12 Picture of a solution of **Cu(tmpa)** without (left, blue) and with added $NaNO_2$ (green, right). Conditions: 2.0 mM **Cu(tmpa)**, 250 mM $NaNO_2$, 0.1 M PB pH 7 (changed to pH 6.8 upon addition of 250 mM $NaNO_2$).

8. EPR spectra of Cu(tmpa) solutions in the presence of nitrite

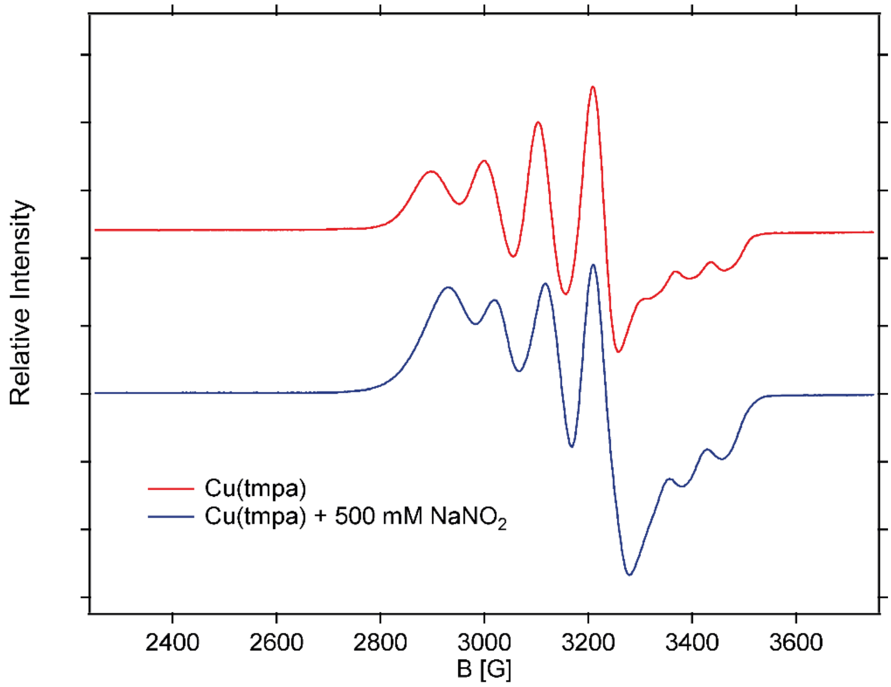

Figure S13 EPR spectra of 1.0 mM Cu(tmpa) in absence (red line) and presence of 500 mM $NaNO_2$ (blue line) recorded in a 9:1 mixture of H_2O and glycerol at 140 K.

9. DFT calculations

DFT calculations as well as visualization and structural analysis of the calculated structures were performed using the Amsterdam Density Functional (ADF) engines of the AMS2022 program package developed by SCM.[5, 6] All DFT calculations were performed using the B3LYP exchange correlation functional[7, 8] including D4 dispersion corrections.[9] A triple zeta basis set with a polarization function (TZP)[10] was used and solvent effects in water were accounted for using the COSMO implicit solvent model.[11] The geometry that formed the starting point of the DFT calculations was obtained from the previously reported crystal structure of $[Cu(tmpa)(NO_2\text{-}\kappa N)]^+$.[12] Thermodynamic corrections to the electronic energies were calculated using the harmonic-oscillator model at P = 1 atm and T = 298 K. **Table S1** shows for all optimized geometries in this study the overall charge of the structure, its spin multiplicity and the calculated Gibbs Free Energy in kcal/mol.

Table S1 Gibbs Free Energies calculated at 298.15 K in kcal/mol at B3LYP level of theory with the D4EEQ dispersion correction and a TZP basis set using COMSO as implicit solvent model for water

Entry	Compound	Charge	Spin multiplicity	Gibbs Free Energy (kcal/mol)	Figure
1	$[Cu(tmpa)(NO_2\text{-}\kappa N)]^+$	1	Doublet	-7206.73	S14
2	$[Cu(tmpa)(NO_2\text{-}\kappa O)]^+$	1	Doublet	-7206.95	S15
3	$[Cu(tmpa)(NO_2\text{-}\eta_2\text{-}O,O)]^+$	1	Doublet	-7204.85	S16
4	$[Cu(tmpa)(NO_2\text{-}\kappa N)]$	0	Singlet	-7295.68	S17
5	$[Cu(tmpa)(NO_2\text{-}\kappa O)]$	0	Singlet	-7293.62	S18
6	$[Cu(tmpa)(NO_2\text{-}\eta_2\text{-}O,O)]$	0	Singlet	-7293.37	S19
7	$[Cu(tmpa)(HNO_2\text{-}\kappa N)]$	1	Singlet	-7288.74	S20
8	$[Cu(tmpa)(HNO_2\text{-}\kappa O)]$	1	Singlet	-7285.65	S21
9	$[Cu(tmpa)(H_2NO_2\text{-}\kappa N)]$	2	Singlet	-7289.83	S22
10	$[Cu(tmpa)(HONOH\text{-}\kappa N)]$	2	Singlet	-7250.61	S23
11	$[Cu(tmpa)(HNO_2\text{-}\kappa N)]\text{-}H_2PO_4$ TS	0	Singlet	-8581.12	S24
12	$[Cu(tmpa)(HNO_2\text{-}\kappa N)]^+ + H_2PO_4^-$	0	Singlet	-8586.88	S25
13	$[Cu(tmpa)(NO\text{-}\kappa N)]^{2+} + HPO_4^{2-} + H_2O$	0	Singlet	-8580.25	S26
14	$[Cu(tmpa)(H_2O)]^{2+} + HPO_4^{2-} +$ NO	0	Triplet	-8589.78	S27
15	$[Cu(tmpa)(HPO_4)]$ + NO + H_2O	0	Triplet	-8595.94	S28

9.1 Binding of nitrite to Cu(II)tmpa

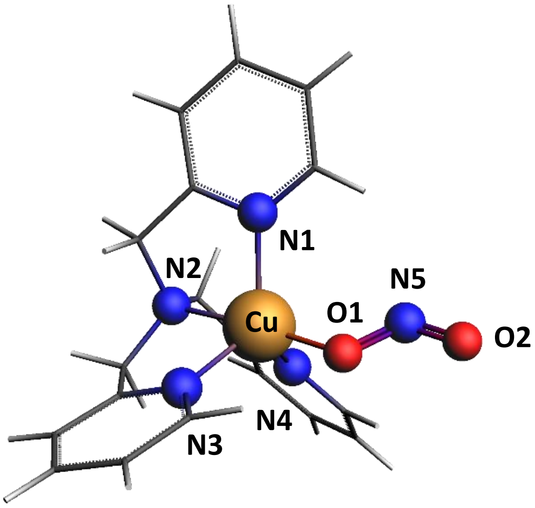

Figure S14 Geometry optimized structure of **[Cu(tmpa)(NO_2-κO)]$^+$** at B3LYP-D4 level of theory with charge = 1, spin multiplicity = 1. Selected bond distances (Å): N1-Cu 2.088, N2-Cu 2.056, N3-Cu 2.080, N4-Cu 2.091, O1-Cu 1.943, O1-N5 1.307, O2-N5 1.228 and angles (°): O1-N5-O2 113.7.

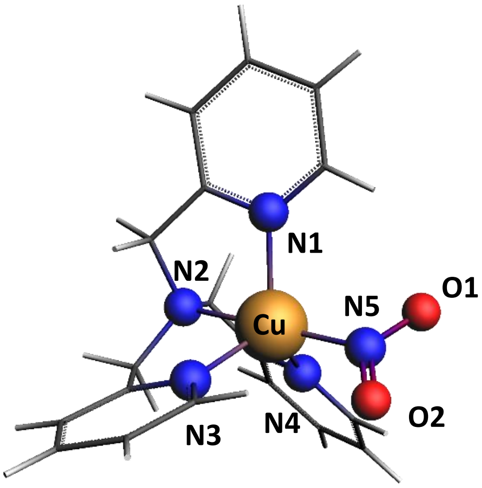

Figure S15 Geometry optimized structure of **[Cu(tmpa)(NO_2-κN)]$^+$** at B3LYP-D4 level of theory with charge = 1, spin multiplicity = 1. Selected bond distances (Å): N1-Cu 2.100, N2-Cu 2.066, N3-Cu 2.101, N4-Cu 2.083, N5-Cu 1.992, O1-N5 1.245, O2-N5 1.242 and angles (°): O1-N5-O2 120.2.

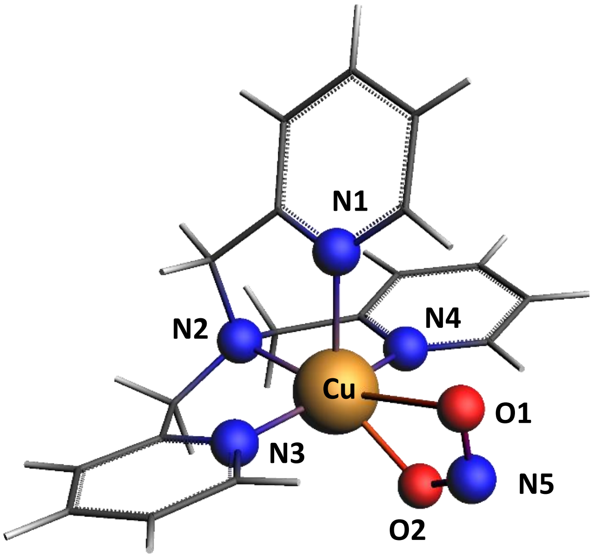

Figure S16 Geometry optimized structure of **[Cu(tmpa)(NO_2-η_2-O,O)]$^+$** at B3LYP-D4 level of theory with charge = 1, spin multiplicity = 1. Selected bond distances (Å): N1-Cu 2.275, N2-Cu 2.113, N3-Cu 1.993, N4-Cu 2.001, O1-Cu 2.852, O2-Cu 2.010, O1-N5 1.234, O2-N5 1.291 and angles (°): O1-N5-O2 115.4, N3-Cu-N4 161.7.

9.2 Binding of nitrite to Cu(I)tmpa

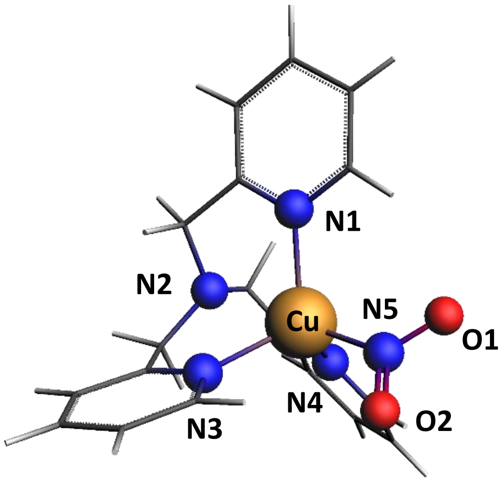

Figure S17 Geometry optimized structure of **[Cu(tmpa)(NO_2-κN)]** at B3LYP-D4 level of theory with charge = 0, spin multiplicity = 0. Selected bond distances (Å): N1-Cu 2.131, N2-Cu 2.490, N3-Cu 2.150, N4-Cu 2.117, N5-Cu 2.050, O1-N5 1.263, O2-N5 1.261 and angles (°): O1-N5-O2 116.4.

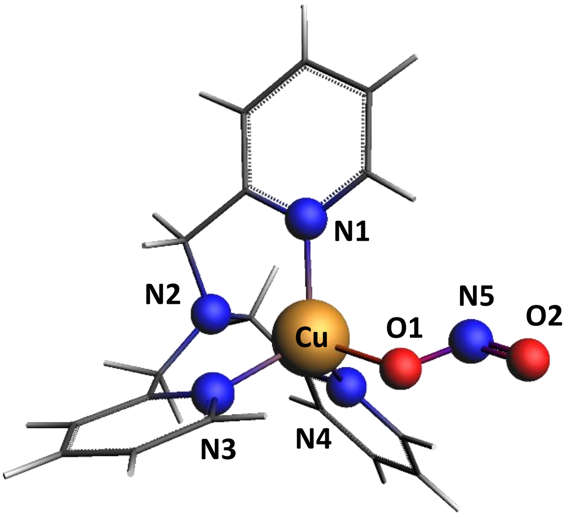

Figure S18 Geometry optimized structure of **[Cu(tmpa)(NO_2-κO)]** at B3LYP-D4 level of theory with charge = 0, spin multiplicity = 0. Selected bond distances (Å): N1-Cu 2.069, N2-Cu 2.419, N3-Cu 2.093, N4-Cu 2.066, O1-Cu 2.205, O1-N5 1.274, O2-N5 1.258 and angles (°): O1-N5-O2 114.9.

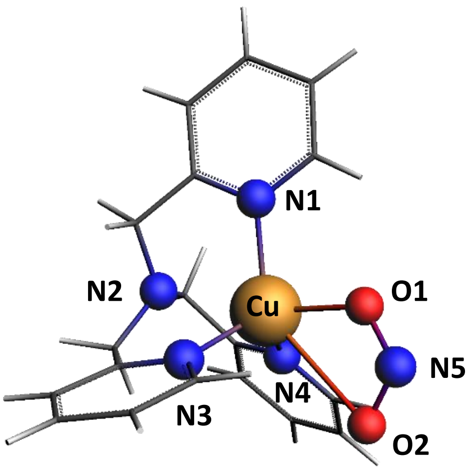

Figure S19 Geometry optimized structure of **[Cu(tmpa)(NO_2-η$_2$-O,O)]** at B3LYP-D4 level of theory with charge = 0, spin multiplicity = 0. Selected bond distances (Å): N1-Cu 2.128, N2-Cu 2.456, N3-Cu 2.091, N4-Cu 2.075, O1-Cu 2.179, O2-Cu 3.007, O1-N5 1.276, O2-N5 1.253 and angles (°): O1-N5-O2 115.7.

9.3 Binding of HNO_2 to Cu(I)tmpa

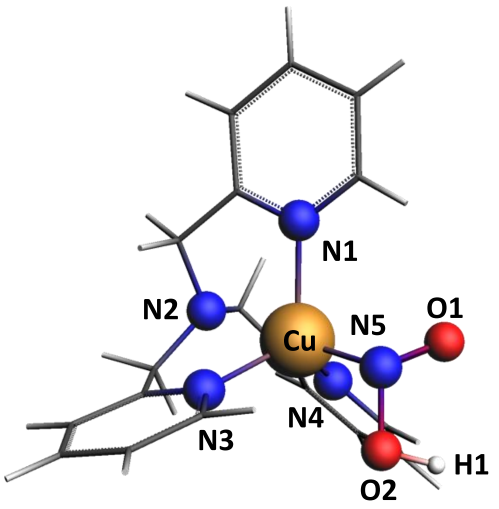

Figure S20 Geometry optimized structure of **[Cu(tmpa)(HNO_2-κN)]$^+$** at B3LYP-D4 level of theory with charge = 1, spin multiplicity = 0. Selected bond distances (Å): N1-Cu 2.147, N2-Cu 2.209, N3-Cu 2.118, N4-Cu 2.033, N5-Cu 2.002, O1-N5 1.216, O2-N5 1.436, O2-H1 0.980 and angles (°): O1-N5-O2 112.1.

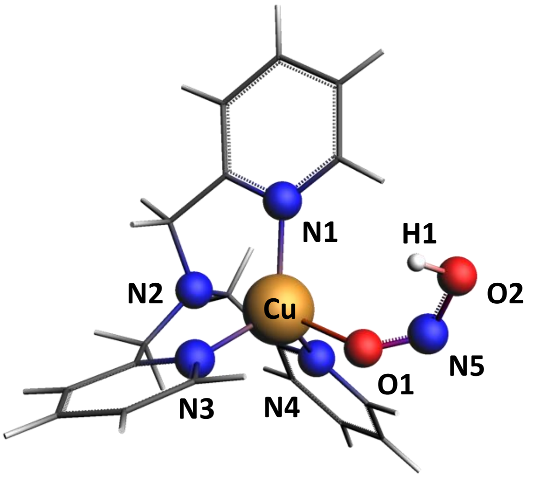

Figure S21 Geometry optimized structure of **[Cu(tmpa)(HNO_2-κO)]$^+$** at B3LYP-D4 level of theory with charge = 1, spin multiplicity = 0. Selected bond distances (Å): N1-Cu 2.060, N2-Cu 2.231, N3-Cu 2.049, N4-Cu 2.071, N5-Cu 2.234, O1-N5 1.208, O2-N5 1.400, O2-H1 0.983 and angles (°): O1-N5-O2 113.0.

9.4 Second protonation step

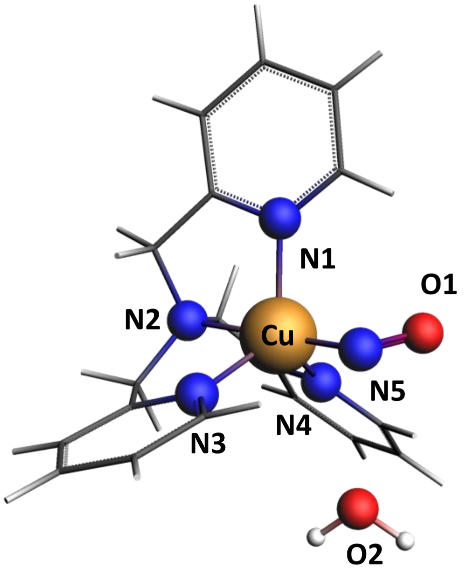

Figure S22 Geometry optimized structure of **[Cu(tmpa)(H_2NO_2-κN)]$^{2+}$** at B3LYP-D4 level of theory with charge = 2, spin multiplicity = 0. Selected bond distances (Å): N1-Cu 2.046, N2-Cu 2.055, N3-Cu 2.044, N4-Cu 2.030, N5-Cu 1.881, O1-N5 1.136, and angles (°): Cu-N5-O1 119.3.

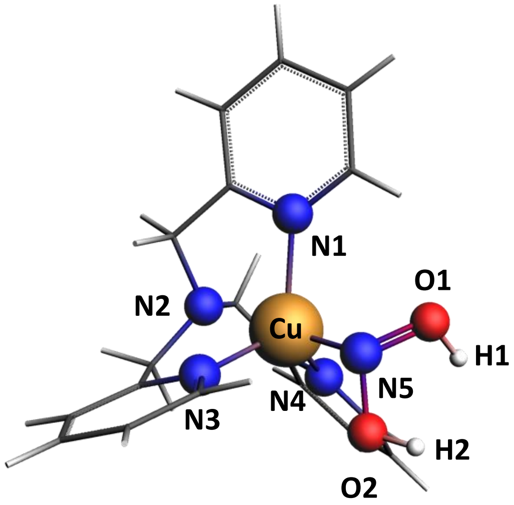

Figure S23 Geometry optimized structure of **[Cu(tmpa)(HONHO-κN)]$^{2+}$** at B3LYP-D4 level of theory with charge = 2, spin multiplicity = 0. Selected bond distances (Å): N1-Cu 2.117, N2-Cu 2.023, N3-Cu 2.053, N4-Cu 2.014, N5-Cu 1.903, O1-N5 1.362, O2-N5,1.386, O1-H1 0.982, O2-H2 0.979 and angles (°): O1-N5-O2 109.3.

9.5 Energy profile of the RDS

In order to identify a transition state structure belonging to the rate-determining, second protonation step, a linear transit calculation was carried out. In this calculation, the distance between a proton on a $H_2PO_4^-$ molecule and the oxygen atom of HNO_2, bound to the copper centre, was changed from 1.97 to 0.98 Å. This calculation resulted in identification of a transition state structure (See **Figure S24**), which showed only one single mode with a negative frequency. The reactant and product states connected to this transition state where found by an intrinsic reaction coordinate (IRC) calculation, resulting in the structures present in **Figure S25** and **Figure S26**. Analysis of the product state showed that two other local minima could be accessed (**Figure S27** and **Figure S28**) upon changing the spin multiplicity of the system to the triplet state.

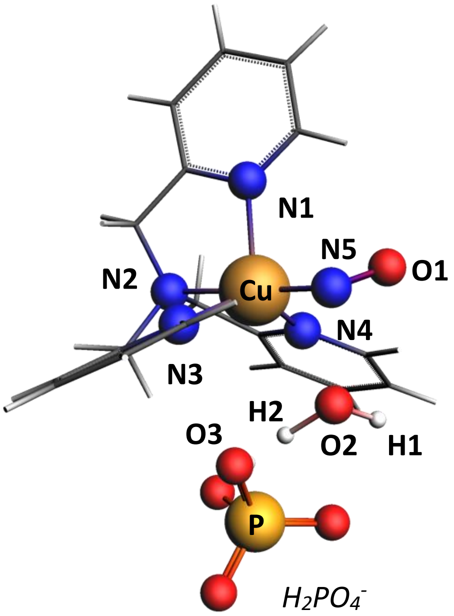

Figure S24 Transition state geometry of **[Cu(tmpa)(HNO_2-κN)]$^+$ + $H_2PO_4^-$** at B3LYP-D4 level of theory with charge = 0, spin multiplicity = 0. Selected bond distances (Å): N1-Cu 2.140, N2-Cu 2.083, N3-Cu 2.019, N4-Cu 2.012, N5-Cu 1.923, O1-N5 1.149, O2-N5 2.125, O2-H1 0.970, O2-H2 1.192, O3-H2 1.231 and angles (°): O1-N5-O2 117.6.

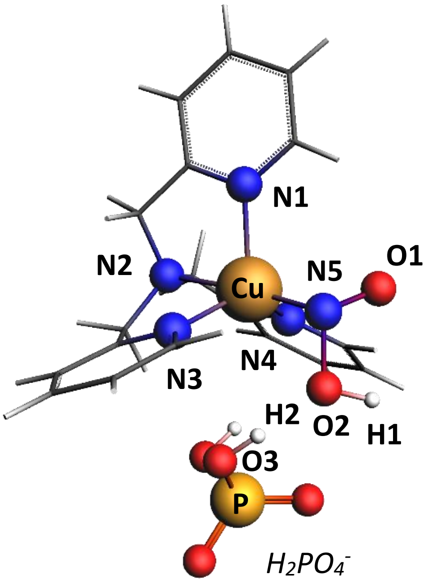

Figure S25 Geometry optimized structure of **[Cu(tmpa)(HNO$_2$-κN)]$^+$ + H$_2$PO$_4$$^-$** at B3LYP-D4 level of theory with charge = 0, spin multiplicity = 0. Selected bond distances (Å): N1-Cu 2.141, N2-Cu 2.179, N3-Cu 2.076, N4-Cu 2.028, N5-Cu 1.999, O1-N5 1.208, O2-N5 1.500, O2-H1 0.978, O2-H2 1.898, O3-H2 0.978 and angles (°): O1-N5-O2 111.3.

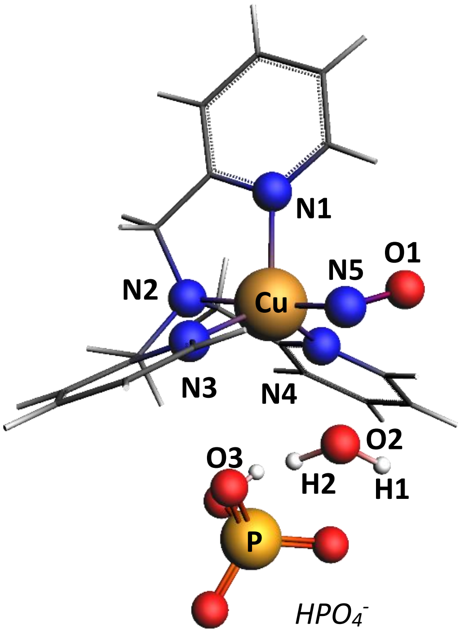

Figure S26 Geometry optimized structure of **[Cu(tmpa)(NO-κN)]$^{2+}$ + H$_2$O + HPO$_4$$^{2-}$** at B3LYP-D4 level of theory with charge = 0, spin multiplicity = 0. Selected bond distances (Å): N1-Cu 2.116, N2-Cu 2.063, N3-Cu 2.014, N4-Cu 2.013, N5-Cu 1.897, O1-N5 1.141, O2-N5 2.412, O3-H2 1.518 and angles (°): Cu-N5-O1 118.3.

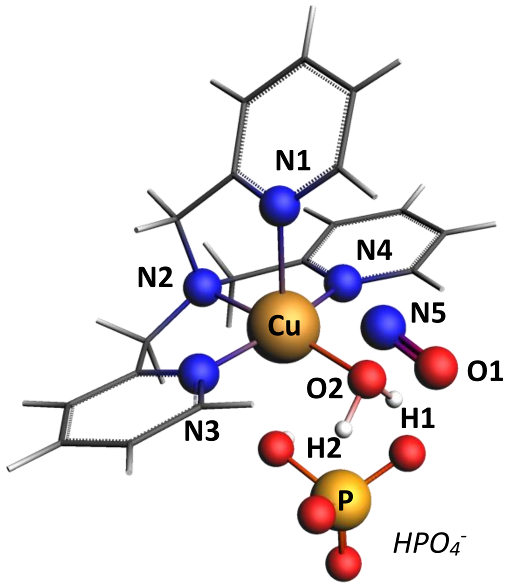

Figure S27 Geometry optimized structure of **[Cu(tmpa)(H_2O)]$^{2+}$ + NO + HPO_4^{2-}** at B3LYP-D4 level of theory with charge = 0, spin multiplicity = 0. Selected bond distances (Å): N1-Cu 2.343, N2-Cu 2.066, N3-Cu 1.997, N4-Cu 2.013, O2-Cu 1.991, O2-H1 1.028, O2-H2 1.003, O1-N5 1.154.

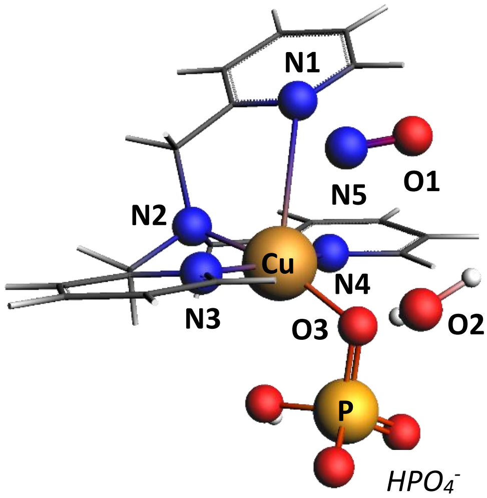

Figure S28 Geometry optimized structure of **[Cu(tmpa)(HPO_4)] + NO + H_2O** at B3LYP-D4 level of theory with charge = 0, spin multiplicity = 0. Selected bond distances (Å): N1-Cu 3.123, N2-Cu 2.068, N3-Cu 1.990, N4-Cu 1.980, O3-Cu 1.952, O1-N5 1.159.

9.6 XYZ coordinates of optimized structures

Structure: [Cu(tmpa)(NO_2-κN)]$^+$
Gibbs free energy: -7206.73 kcal/mol
Figure: S14

Cu	4.091484456	1.986105405	5.922083479
C	4.769036809	-0.333199984	7.850492944
H	3.869457087	-0.032509047	8.36924026
C	5.504230575	-1.430277054	8.269164671
H	5.179311742	-1.997989968	9.129501279
C	6.643155904	-1.782077148	7.555086083
H	7.232593808	-2.640492648	7.848262319
C	7.012170474	-1.022752252	6.451914678
H	7.885999393	-1.272414874	5.866198123
C	6.233095927	0.068955523	6.100584845
C	6.583302441	0.973222839	4.949370988
H	7.178911491	1.807982773	5.322235552
H	7.180073636	0.448780166	4.201085582
C	4.638275295	0.535864688	3.515755127
H	4.70599481	-0.431519057	4.015225959
H	5.110511589	0.43647542	2.536882119
C	3.189431637	0.92228711	3.387606424
C	2.439872615	0.618230261	2.261572655
H	2.903221605	0.112202909	1.425751397
C	1.098842518	0.97953368	2.231793944
H	0.491993278	0.753523813	1.364930285
C	0.554220343	1.644414533	3.323205886
H	-0.482234652	1.951303227	3.336685633
C	1.368261943	1.920106111	4.410880392
H	0.99182275	2.429414266	5.285623461
C	5.613476037	2.78192483	3.605675879
H	4.773680519	2.952605648	2.930713151
H	6.511315674	2.688870618	2.992110366
C	5.714263792	3.951510066	4.546973693
C	6.450150814	5.083292672	4.228132394
H	7.015929914	5.1160405	3.307210243
C	6.439771686	6.158908937	5.10591682
H	7.001383483	7.055118432	4.877673749
C	5.704640011	6.064159317	6.28152649
H	5.672387334	6.876482721	6.994203609
C	5.009390743	4.894226139	6.540422865
H	4.438829618	4.762917689	7.448519139
N	5.127610551	0.401913968	6.790251458
N	5.350929198	1.530338726	4.34949715
N	2.658296671	1.561950038	4.445269097
N	5.010586044	3.859468231	5.688822458
O	3.462877396	2.887480837	8.503250782
N	2.942288654	2.352896762	7.507194857
O	1.752825794	1.997630887	7.539890764

Structure: $[Cu(tmpa)(NO_2\text{-}\kappa O)]^+$
Gibbs free energy: -7206.95 kcal/mol
Figure: S15

Cu	4.03692092	2.066427018	5.868665769
C	4.664165236	-0.207567268	7.876202526
H	3.7621724	0.124702347	8.371080835
C	5.373839983	-1.308501288	8.329856017
H	5.028502471	-1.850889448	9.198560397
C	6.515317007	-1.696959367	7.638753074
H	7.085625793	-2.558541433	7.959371343
C	6.912232734	-0.971069108	6.522386079
H	7.789193403	-1.250047477	5.954788996
C	6.157151392	0.125830169	6.13680788
C	6.537199046	1.000885725	4.971728112
H	7.141461991	1.832471019	5.336100757
H	7.133955737	0.453575658	4.240538505
C	4.624475083	0.57287385	3.492196948
H	4.714711282	-0.400304671	3.97605296
H	5.100127329	0.498982519	2.513044084
C	3.164404592	0.919226417	3.370330485
C	2.401037633	0.559507883	2.270115862
H	2.862492575	0.051044554	1.434723631
C	1.046082464	0.867285559	2.267514545
H	0.428959011	0.598400264	1.420493777
C	0.49808087	1.532146882	3.358287779
H	-0.550213379	1.793848284	3.390867696
C	1.32698714	1.86886915	4.416840734
H	0.955674945	2.391936091	5.287370585
C	5.610373078	2.817868343	3.603079805
H	4.764223501	3.016905621	2.944183899
H	6.496109209	2.703283564	2.976045087
C	5.760175255	3.970044886	4.559810698
C	6.556504358	5.068554764	4.274926644
H	7.132659777	5.094565752	3.360377987
C	6.596092197	6.119765909	5.182124812
H	7.20808023	6.989392521	4.982450658
C	5.847665495	6.034970069	6.35003515
H	5.854809681	6.828257607	7.084324505
C	5.087055078	4.898224647	6.571770778
H	4.500743185	4.777985018	7.471747541
N	5.050532874	0.495032506	6.804514241
N	5.32099349	1.567549909	4.342656178
N	2.630367341	1.565095896	4.420326163
N	5.041626786	3.888929862	5.693298357
N	3.188943219	2.875298484	8.349380778
O	2.691007571	2.473316372	7.210230734

O	2.338797978	3.105304924	9.205282564

Structure: $[Cu(tmpa)(NO_2\text{-}\eta_2\text{-}O,O)]^+$
Gibbs free energy: -7204.85 kcal/mol
Figure: S16

Cu	3.819765426	0.937841991	5.58147998
C	5.588399092	0.229787483	7.865070865
H	4.682891785	0.344465711	8.443640848
C	6.816153526	0.02767061	8.471326822
H	6.882012136	-0.021649851	9.548763959
C	7.943605887	-0.093721973	7.667423999
H	8.920036771	-0.239747848	8.109546045
C	7.805916342	-0.019883258	6.287295934
H	8.66249506	-0.108214775	5.633402878
C	6.54370734	0.173352695	5.747602398
C	6.292987271	0.187710426	4.261239025
H	7.184304442	0.510454687	3.722438947
H	6.07452516	-0.833322968	3.948241157
C	4.314182783	0.507614683	2.800066953
H	4.287795274	-0.578848735	2.881717661
H	4.750914885	0.759110609	1.8321827
C	2.906754644	1.035927304	2.909081866
C	2.100597991	1.260955011	1.805340113
H	2.491542475	1.093591806	0.81146312
C	0.798243791	1.704129701	2.002392557
H	0.151166987	1.886758605	1.15483337
C	0.341813487	1.918932835	3.297222812
H	-0.661700327	2.269532261	3.491739445
C	1.204557584	1.682316064	4.354353315
H	0.90296299	1.843573496	5.378898147
C	5.501721965	2.450412311	3.682051403
H	4.714896651	2.900039039	3.075943524
H	6.426620945	2.499863529	3.10590579
C	5.616710455	3.251391786	4.953875772
C	6.613153273	4.201049632	5.132733086
H	7.369697288	4.344008265	4.373351965
C	6.614725133	4.954935046	6.301436734
H	7.380576502	5.700547432	6.469084559
C	5.624621576	4.730718648	7.249850893
H	5.591083695	5.292579822	8.173064508
C	4.673573745	3.750347939	6.996946443
H	3.894125724	3.523420981	7.7112334
N	5.459929034	0.300126209	6.533705157
N	5.125267571	1.032919619	3.92289876
N	2.457081128	1.249551987	4.160411511
N	4.668133193	3.02796736	5.873083649
O	2.56338151	-0.016507728	6.827341717
N	1.925759743	0.680247256	7.707919744
O	2.155464328	1.892139342	7.692432124

Structure: [Cu(tmpa)(NO_2-κN)]
Gibbs free energy: -7295.68 kcal/mol
Figure: S17

Cu	3.924897187	2.065514524	6.106004311
C	4.82323287	-0.250708399	7.852625612
H	3.953566109	0.082087072	8.405461484
C	5.529714048	-1.375444936	8.251127236
H	5.213419503	-1.931904939	9.122882084
C	6.633594938	-1.763385373	7.500907065
H	7.20675101	-2.640354078	7.772555511
C	6.9824025	-1.009648608	6.388170822
H	7.828466183	-1.285229177	5.772242617
C	6.227994319	0.110941969	6.058138867
C	6.607883491	0.983842584	4.880456856
H	7.187241664	1.828509513	5.261741721
H	7.272249936	0.422762008	4.210135733
C	4.679868248	0.544406441	3.436939037
H	4.765085611	-0.423483893	3.936844808
H	5.066347241	0.407851768	2.4185914
C	3.211074325	0.906876823	3.376037518
C	2.463073296	0.662778855	2.229472603
H	2.945471643	0.247667236	1.354129209
C	1.106480794	0.960930824	2.224368722
H	0.506908482	0.779260918	1.341507423
C	0.538511671	1.501807438	3.371433244
H	-0.512743385	1.753020806	3.417671297
C	1.353834917	1.726569165	4.471033402
H	0.958429615	2.141309553	5.389201173
C	5.638564839	2.789805663	3.545758991
H	4.772890398	2.97639165	2.905009551
H	6.51994256	2.799551603	2.891250543
C	5.73356434	3.931931334	4.534389036
C	6.548663293	5.027983825	4.271940835
H	7.155382692	5.040349789	3.375839845
C	6.571900883	6.089537374	5.166020594
H	7.200114348	6.951186403	4.979933885
C	5.780358875	6.018367499	6.306593968
H	5.76577337	6.817315219	7.035854154
C	5.005025452	4.886088403	6.505351886
H	4.382208997	4.776572814	7.38432116
N	5.157670923	0.482256229	6.780948331
N	5.437604319	1.514138986	4.206271603
N	2.663480331	1.440451559	4.480673486
N	4.976739488	3.859371807	5.642072343
O	3.060329197	3.227197013	8.63270238
N	2.678649292	2.497518996	7.674771692
O	1.501051598	2.050913454	7.745005008

Structure: [Cu(tmpa)(NO_2-κO)]
Gibbs free energy: -7293.62 kcal/mol
Figure: S18

Cu	3.973232056	2.093487122	5.996831544
C	4.805726963	-0.08501087	7.903648568
H	3.961866924	0.307075701	8.456325172
C	5.489437122	-1.20815884	8.344586114
H	5.180505432	-1.706970265	9.252783057
C	6.561539846	-1.669934572	7.590121583
H	7.115315381	-2.548032413	7.895178602
C	6.905947358	-0.986436126	6.431047532
H	7.730628372	-1.318299613	5.814078333
C	6.179415777	0.138534891	6.058789205
C	6.57146526	0.94357616	4.834203518
H	7.192682682	1.77709575	5.171370145
H	7.201913122	0.32633608	4.181404745
C	4.618230424	0.549455228	3.398866267
H	4.714656984	-0.424976552	3.884000203
H	4.963750453	0.419762394	2.365206719
C	3.146287981	0.916098106	3.393100893
C	2.351029718	0.642229243	2.286104788
H	2.797806728	0.207644677	1.401517251
C	0.993623594	0.933591062	2.330507959
H	0.359137949	0.727695103	1.478315181
C	0.470275154	1.50006364	3.486949081
H	-0.578878956	1.748884262	3.570012067
C	1.329598592	1.756467011	4.544797265
H	0.969701347	2.203371214	5.46240066
C	5.632927532	2.781751716	3.504177223
H	4.762278818	2.988457275	2.876726307
H	6.506913648	2.787810317	2.840051639
C	5.758424687	3.912095493	4.506467982
C	6.613082566	4.981392836	4.26542864
H	7.226582232	4.984161308	3.37414316
C	6.669476934	6.029501197	5.174977396
H	7.330251152	6.869416853	5.005049999
C	5.869227604	5.971833512	6.310115782
H	5.880511013	6.759809856	7.050701098
C	5.051005518	4.86663862	6.487970692
H	4.421596305	4.770888164	7.363474842
N	5.13569415	0.578800999	6.785395113
N	5.418331991	1.499384445	4.14971268
N	2.640348228	1.474776524	4.50686877
N	4.990815844	3.853581538	5.610448738
N	2.935867938	3.043766147	8.621997806
O	2.466540037	2.5886055	7.528956196
O	2.072545503	3.318259293	9.49443544

Structure: [Cu(tmpa)(NO_2-η_2-O,O)]
Gibbs free energy: -7293.37 kcal/mol
Figure: S19

Cu	4.007296575	1.754605177	6.053326293
C	5.191145537	-0.525429236	7.618220779
H	4.31543809	-0.316443041	8.218010857
C	6.029481924	-1.59059399	7.910804792
H	5.819985982	-2.223237628	8.762935427
C	7.119589745	-1.825010963	7.081216233
H	7.791459666	-2.652905589	7.267462465
C	7.327199704	-0.978621652	6.00025107
H	8.158817456	-1.131863836	5.324508338
C	6.452124755	0.079525479	5.780206877
C	6.698842419	1.057102979	4.647963886
H	7.255966857	1.903229838	5.057591122
H	7.350449499	0.586927075	3.900059765
C	4.731921944	0.637489531	3.24729171
H	4.929811072	-0.371148646	3.618507492
H	5.056056174	0.655976826	2.19844389
C	3.234491288	0.863332129	3.31190123
C	2.434786307	0.628600333	2.198784174
H	2.893781048	0.331596184	1.264688756
C	1.058109224	0.778182826	2.302163214
H	0.419770672	0.598611653	1.446619512
C	0.520092993	1.17142341	3.52203929
H	-0.544912912	1.305314114	3.654435594
C	1.384028415	1.399825793	4.582474922
H	1.009221757	1.709017692	5.549335928
C	5.522830591	2.933005206	3.592576766
H	4.596132827	3.130807838	3.048076312
H	6.346822633	3.101426873	2.887109889
C	5.616663769	3.933096052	4.726863071
C	6.394409586	5.079322775	4.608006256
H	6.972323608	5.243157662	3.707571928
C	6.422043435	5.99619594	5.651528368
H	7.023737996	6.892968572	5.578575035
C	5.67046173	5.733772725	6.79022615
H	5.661035235	6.414136872	7.631269909
C	4.927756041	4.562537917	6.837227719
H	4.330741522	4.312838099	7.704831924
N	5.390105508	0.29871349	6.576940588
N	5.463854989	1.568467323	4.084514112
N	2.713852637	1.252379244	4.489215605
N	4.896928469	3.675169163	5.833199491
O	1.986554891	0.359663543	7.789355997
N	1.888615627	1.538911641	8.200133719

O	2.712329014	2.372017668	7.693569246

Structure: [Cu(tmpa)(HNO_2-κN)]
Gibbs free energy: -7288.74 kcal/mol
Figure: S20

Cu	3.999914267	1.981558488	5.941219639
C	4.780600159	-0.148970796	7.942352188
H	3.907084169	0.196625155	8.477727679
C	5.519650721	-1.222523031	8.41152446
H	5.225272665	-1.719852255	9.324953623
C	6.623694638	-1.639345552	7.678374583
H	7.218383382	-2.481678187	8.006227158
C	6.94976962	-0.96095226	6.511870597
H	7.79924178	-1.258338215	5.912053896
C	6.170973421	0.115887374	6.111286825
C	6.533270485	0.933369215	4.893562113
H	7.162585148	1.762696583	5.222947175
H	7.130364487	0.334590827	4.19947995
C	4.599823887	0.533200932	3.441000546
H	4.663606302	-0.437761186	3.936332465
H	5.031861312	0.413727618	2.442677944
C	3.142117609	0.921373882	3.338562406
C	2.381397515	0.608923978	2.219559837
H	2.841057832	0.10434599	1.380205024
C	1.037252942	0.9583278	2.197939884
H	0.425247902	0.723323907	1.336908567
C	0.496628857	1.625468701	3.290929071
H	-0.541169121	1.928305733	3.311880805
C	1.323577219	1.910711604	4.366535288
H	0.950766336	2.435544334	5.236455208
C	5.608995549	2.7633624	3.557942513
H	4.756445077	2.973157288	2.909255619
H	6.492411627	2.701066803	2.915059
C	5.751386761	3.910628439	4.53219607
C	6.56971665	4.99632392	4.246537599
H	7.153182413	5.004374895	3.335595111
C	6.624160263	6.055934671	5.142248988
H	7.254703424	6.911968385	4.939979286
C	5.864898301	5.99279309	6.30469286
H	5.879771294	6.79133346	7.033833617
C	5.082814435	4.86935603	6.525031099
H	4.485420754	4.769555315	7.421343989
N	5.093086724	0.508959996	6.817962987
N	5.343429786	1.50119448	4.25425059
N	2.61430587	1.560055812	4.395849994
N	5.022686857	3.848178021	5.659470549
O	3.127587357	2.914908019	8.40210599
N	2.677082506	2.498191622	7.351850513
O	1.797241905	1.379909002	7.548144052
H	1.745700907	1.274423382	8.5214325

Structure: [Cu(tmpa)(HNO_2-κO)]
Gibbs free energy: -7285.65 kcal/mol
Figure: S21

Cu	4.029371159	1.987498378	5.928106707
C	4.835103937	-0.20729619	7.90985895
H	3.97535459	0.143522466	8.464558674
C	5.555326265	-1.312928163	8.335418452
H	5.258986954	-1.836398752	9.233882075
C	6.644463177	-1.726438277	7.578265928
H	7.225409393	-2.590655392	7.872542384
C	6.971917998	-1.016379647	6.430360482
H	7.80841947	-1.312973092	5.811721731
C	6.209945246	0.088624433	6.073404187
C	6.57247715	0.929738724	4.865874674
H	7.196757946	1.75711435	5.210411145
H	7.179899255	0.340844801	4.170835527
C	4.621070426	0.558776997	3.416982018
H	4.736252357	-0.43223492	3.861377696
H	4.991000961	0.491098248	2.388583899
C	3.144209395	0.896625518	3.412203859
C	2.346786433	0.613469654	2.311312497
H	2.793993409	0.192022384	1.420870881
C	0.98462522	0.87859098	2.370775293
H	0.347399367	0.664953332	1.522519016
C	0.458307239	1.427855505	3.53359632
H	-0.594977134	1.654637736	3.625653967
C	1.3185067	1.696723554	4.58704842
H	0.954016098	2.132330385	5.508111699
C	5.613420542	2.798495284	3.591857538
H	4.752944551	3.009420112	2.952899402
H	6.497576746	2.801818718	2.945736809
C	5.720499191	3.911920693	4.612099628
C	6.532505659	5.0133321	4.375454982
H	7.128260405	5.053062664	3.473382242
C	6.569062649	6.04578981	5.302845617
H	7.19522162	6.912328233	5.135012141
C	5.796874882	5.938715933	6.452630325
H	5.795924742	6.712229779	7.208315088
C	5.02238319	4.802972935	6.628020113
H	4.422925566	4.679116512	7.518760831
N	5.150991831	0.483085187	6.80549174
N	5.388845957	1.501996888	4.227553899
N	2.632342776	1.439167998	4.533468508
N	4.974664009	3.806606119	5.730298518
N	2.791628453	2.506888989	8.660252762
O	2.488008578	2.197627623	7.532169109

O	2.50239129	3.844097706	8.958228849
H	2.033535397	4.209078878	8.175444921

Structure: [Cu(tmpa)(H_2NO_2-κN)]
Gibbs free energy: -7289.83 kcal/mol
Figure: S22

Cu	4.102070748	2.114892912	5.801569857
C	4.752465663	0.080704027	7.974772493
H	3.874338471	0.462195032	8.474892288
C	5.481248285	-0.963479752	8.517453641
H	5.177208157	-1.395381368	9.460062235
C	6.583849432	-1.440328856	7.819856802
H	7.167205654	-2.263664013	8.209258384
C	6.925833835	-0.850455749	6.610316241
H	7.774603806	-1.197342843	6.037744178
C	6.1629835	0.204087403	6.135459801
C	6.527304271	0.945223873	4.87613776
H	7.203165358	1.759476305	5.139756135
H	7.054820753	0.29727956	4.175310214
C	4.556003096	0.563312361	3.447976577
H	4.61928966	-0.403298458	3.949599424
H	4.986518601	0.446644274	2.452500166
C	3.111540862	0.981712	3.375587646
C	2.288577736	0.667032831	2.306852498
H	2.68956597	0.131204779	1.457936428
C	0.954198104	1.053221933	2.350113798
H	0.293498306	0.818338698	1.526449965
C	0.483008976	1.7532331	3.454234997
H	-0.545553986	2.07714113	3.5221486
C	1.366422592	2.043062683	4.481600014
H	1.052028787	2.589500016	5.361519177
C	5.623715006	2.782766632	3.50958571
H	4.765230871	3.008918424	2.876187924
H	6.488930812	2.653821877	2.857540761
C	5.835707732	3.921795086	4.471280009
C	6.671660981	4.987432054	4.180402266
H	7.222193203	4.9977587	3.250219856
C	6.789712591	6.02344197	5.098210929
H	7.435046723	6.866049965	4.88944813
C	6.080418271	5.956179795	6.290508524
H	6.14917988	6.73513541	7.036182714
C	5.273290146	4.855012793	6.521222072
H	4.718455427	4.763607049	7.444638931
N	5.087144016	0.655497815	6.812001065
N	5.324349007	1.538439498	4.252785687
N	2.64555684	1.660465828	4.441403995
N	5.147869086	3.864980951	5.63010468
O	3.206928052	2.957640375	8.126595408
N	2.856047668	2.72313957	7.072143819
O	1.487047303	0.443976458	7.287788856

H	1.258958392	0.037829507	8.136882527
H	1.620056948	-0.310300593	6.695959708

Structure: [Cu(tmpa)(HONOH-κN)]
Gibbs free energy: -7250.61 kcal/mol
Figure: S23

Cu	4.045519726	1.965104522	5.861685793
C	4.649750744	-0.204186696	7.890500627
H	3.710867533	0.077594466	8.344547328
C	5.396911032	-1.254529875	8.39621414
H	5.051259349	-1.780695287	9.274094593
C	6.567846657	-1.615468694	7.743577119
H	7.167638662	-2.439984458	8.10426856
C	6.952280579	-0.914262637	6.607968313
H	7.846477694	-1.178372131	6.061359039
C	6.166973486	0.13790565	6.168678015
C	6.532851422	0.973329028	4.977158023
H	7.135338594	1.820539976	5.306060107
H	7.122117951	0.405908793	4.255943235
C	4.595548562	0.49604453	3.522425777
H	4.653294168	-0.459822718	4.043599938
H	5.083349253	0.378628824	2.553958875
C	3.156170178	0.902621107	3.372328688
C	2.38161749	0.567622227	2.274123505
H	2.813839683	0.000203517	1.461880152
C	1.055159454	0.981278175	2.240209327
H	0.429406515	0.730358258	1.394239809
C	0.548051869	1.732193487	3.293647975
H	-0.473603308	2.084130111	3.296554388
C	1.383564074	2.036429432	4.356387935
H	1.043344279	2.624578563	5.19795122
C	5.571177654	2.753844637	3.58415313
H	4.720708421	2.934455764	2.926754478
H	6.454695606	2.633106579	2.955638047
C	5.719584194	3.914542751	4.527227748
C	6.488587617	5.023476561	4.21087571
H	7.038927195	5.049451116	3.280675222
C	6.534606493	6.084330012	5.105874948
H	7.125276384	6.962467085	4.881371753
C	5.821115567	5.995587089	6.294271107
H	5.834187133	6.794198468	7.02269824
C	5.085951515	4.848446684	6.547113533
H	4.528338814	4.733085198	7.464152857
N	5.030442622	0.48597516	6.809617332
N	5.304667176	1.509753537	4.344873587
N	2.652057463	1.618680554	4.394244406
N	5.031043897	3.829378971	5.680320229
O	3.226067597	2.997288996	8.260382848
N	2.707152984	2.477851268	7.113595523
O	2.005251334	1.319976038	7.410549869

H	1.448361331	1.517574281	8.191202303
H	3.342230234	2.284910017	8.926565586

Structure: [Cu(tmpa)(HNO_2-κN)]-H_2PO_4
Gibbs free energy: -8581.12 kcal/mol
Figure: S24

Cu	3.612039281	2.472353067	6.072284563
C	4.164487315	1.16916825	8.738292402
H	3.534902743	1.946281055	9.145591884
C	4.684095354	0.185166291	9.559477952
H	4.452197095	0.1892437	10.61460113
C	5.480780921	-0.799446503	8.992001823
H	5.891433923	-1.595097182	9.598542691
C	5.746642958	-0.748502149	7.629445971
H	6.371724073	-1.491688436	7.153813503
C	5.212509996	0.284828778	6.871488696
C	5.576126319	0.446738436	5.417552761
H	6.544200536	0.947956149	5.377801334
H	5.70423348	-0.527191235	4.942196437
C	3.517937586	0.484025754	4.059153629
H	3.222260896	-0.293519531	4.765758369
H	3.854182531	0.006531601	3.136317352
C	2.322197697	1.363954623	3.814357534
C	1.40668075	1.123493709	2.802390113
H	1.567090205	0.304981748	2.11446605
C	0.289553889	1.942935916	2.698480068
H	-0.442480627	1.769283461	1.921269211
C	0.125318387	2.988411485	3.599423581
H	-0.728526978	3.649129381	3.550525916
C	1.091119409	3.177980912	4.574855122
H	1.013290101	3.974582378	5.304185975
C	5.250601142	2.196090409	3.727328662
H	4.486125487	2.55471165	3.037132239
H	6.007882464	1.679251493	3.134456479
C	5.844577741	3.384213843	4.44273854
C	6.988396728	4.019806485	3.980815907
H	7.502912651	3.637428502	3.109904988
C	7.45346082	5.142914912	4.653927691
H	8.342587405	5.65521955	4.311333501
C	6.76894145	5.588480946	5.77758824
H	7.100125444	6.452779803	6.335748815
C	5.641534094	4.892142266	6.185497673
H	5.09045389	5.199254551	7.064435112
N	4.416587617	1.219555822	7.425549175
N	4.599887079	1.27465739	4.683649697
N	2.157395091	2.383484075	4.674418848
N	5.185321946	3.818423548	5.53202296
O	3.060157117	4.045266034	8.141578462
N	2.574067287	3.656354312	7.176387647
O	1.113947905	2.18572255	7.647215627

H	1.292623	1.935306468	8.566929096
P	1.350355587	-1.214225316	7.161345499
O	1.26420836	0.132169258	6.373037434
O	1.543507952	-0.994396071	8.647694234
O	0.255298155	-2.172621248	6.726601535
O	2.740437476	-1.938232958	6.588782108
H	1.217277803	1.162651978	7.044859855
H	3.492101812	-1.661969734	7.133571007

Structure: $[Cu(tmpa)(HNO_2\text{-}\kappa N)]^+ + H_2PO_4^-$
Gibbs free energy: -8586.88 kcal/mol
Figure: S25

Cu	3.736260873	2.409974683	6.112630962
C	4.242432204	0.63723313	8.523139425
H	3.576757345	1.325122809	9.022681435
C	4.728074775	-0.479788189	9.184655001
H	4.451424643	-0.656521204	10.21513737
C	5.572072952	-1.346007167	8.497375151
H	5.958297852	-2.236720404	8.974473963
C	5.915162514	-1.042728776	7.188361005
H	6.578606934	-1.683035537	6.623268746
C	5.406757253	0.108137347	6.598611719
C	5.858917842	0.536766487	5.222953238
H	6.726317309	1.187268731	5.355747023
H	6.196857801	-0.330874083	4.648504468
C	3.798516069	0.452065594	3.912415835
H	3.59784811	-0.371866892	4.601439461
H	4.129007891	0.010785155	2.966818973
C	2.520365838	1.227952831	3.706983405
C	1.687266107	0.982682902	2.624334549
H	1.960919484	0.231639243	1.895703672
C	0.512766533	1.712644001	2.494708018
H	-0.15330153	1.534319527	1.660807125
C	0.218330535	2.684773976	3.442317487
H	-0.675433439	3.289289355	3.374861145
C	1.102274472	2.875112597	4.493130253
H	0.910201645	3.616846529	5.254548072
C	5.372091933	2.31702529	3.615521722
H	4.572840956	2.624804141	2.939042009
H	6.183943329	1.925928204	2.994990342
C	5.837068825	3.539430365	4.37722571
C	6.913720418	4.299753369	3.938860077
H	7.467960477	3.99226451	3.06212883
C	7.26319224	5.446080455	4.64164944
H	8.09967591	6.051740656	4.318278013
C	6.526165722	5.798038038	5.765912021
H	6.763942754	6.680148652	6.344448695
C	5.47167311	4.981529903	6.146568096
H	4.875988079	5.209797542	7.021237431
N	4.556683716	0.922860183	7.252603464

N	4.827718229	1.299690438	4.521687201
N	2.223684395	2.155054453	4.63579484
N	5.133096755	3.876758885	5.470904467
O	3.169730934	3.48705441	8.634715619
N	2.661470336	3.426200812	7.526888062
O	1.390446974	2.813378874	7.537623462
H	1.302045817	2.200045256	8.34955393
P	0.696041215	-0.366679218	8.21225813
O	1.166752385	-0.009180597	6.695050231
O	1.032404243	0.809531427	9.108842321
O	-0.694546217	-0.916646879	8.201411029
O	1.661376021	-1.627486985	8.563859484
H	1.335549589	0.944658453	6.592970328
H	2.587351112	-1.350811135	8.673192515

Structure: $[Cu(tmpa)(NO\text{-}\kappa N)]^{2+} + HPO_4^{2-} + H_2O$
Gibbs free energy: -8580.25 kcal/mol
Figure: S26

Cu	3.669870986	2.511733337	6.014708785
C	4.127421285	1.129764792	8.660606737
H	3.464153947	1.879012177	9.065569105
C	4.640631167	0.132320438	9.470969795
H	4.379293634	0.105626922	10.51865014
C	5.465052111	-0.828674078	8.901056466
H	5.87167883	-1.631789023	9.500363774
C	5.75381672	-0.752143293	7.543884288
H	6.388888044	-1.48421753	7.064866033
C	5.227239784	0.292585264	6.798470289
C	5.611264597	0.492655286	5.356430804
H	6.56554689	1.020656495	5.339473427
H	5.766815507	-0.465277857	4.858028853
C	3.535221042	0.514330374	4.023085244
H	3.23322897	-0.244415014	4.74842749
H	3.866938755	0.024962741	3.105737018
C	2.33945933	1.392363349	3.782178826
C	1.4308468	1.173961382	2.759490864
H	1.595009369	0.369279398	2.056595841
C	0.318132008	2.00048236	2.661574651
H	-0.40611124	1.846057472	1.873218546
C	0.149130222	3.029351907	3.580508674
H	-0.700660037	3.695304733	3.53518249
C	1.105654396	3.197133866	4.568591071
H	1.024515211	3.981611622	5.310590136
C	5.261239651	2.236558022	3.664343758
H	4.489878757	2.596810441	2.982826895
H	6.011363839	1.717625899	3.065055822
C	5.864494109	3.41693317	4.382263343
C	6.998900319	4.063802416	3.915225472
H	7.499649407	3.699187394	3.029076277
C	7.474327361	5.17285632	4.604085289

H	8.356829472	5.693154513	4.256912159
C	6.810585191	5.594248042	5.749153557
H	7.151100533	6.446468646	6.319772975
C	5.691541857	4.889381682	6.163154368
H	5.157831474	5.178186447	7.058607286
N	4.418427928	1.213078721	7.358068405
N	4.621211177	1.315042804	4.630593795
N	2.168141114	2.39546	4.662246307
N	5.225584185	3.830551102	5.492960405
O	3.157598288	4.059148036	8.090804989
N	2.685734044	3.702261316	7.116608188
O	1.049682723	1.995461348	7.575315731
H	1.108928045	1.411926849	8.365841651
P	1.262596393	-1.207305485	7.500668142
O	1.545158322	-0.267795548	6.316435349
O	1.192013894	-0.454101726	8.834899707
O	0.120337003	-2.190386927	7.262419749
O	2.584446879	-2.236067909	7.601537283
H	1.231665428	1.271457322	6.903362714
H	3.392465587	-1.720577905	7.738326

Structure: $[Cu(tmpa)(H_2O)]^{2+}$ + HPO_4^{2-} + NO
Gibbs free energy: -8589.78 kcal/mol
Figure: S27

Cu	3.244826424	1.670011741	6.029171077
C	4.933096722	1.364911212	8.49203607
H	4.185264494	1.997867663	8.947844248
C	6.070020417	0.981049788	9.181506509
H	6.216970367	1.30906932	10.20040064
C	7.007048527	0.185601215	8.531840132
H	7.910785382	-0.123725512	9.039346428
C	6.77713932	-0.198274893	7.217554928
H	7.489984819	-0.805981738	6.677798981
C	5.611874201	0.218017576	6.592220964
C	5.256180052	-0.19307318	5.187690722
H	6.153886117	-0.388796485	4.599967204
H	4.69037485	-1.122964365	5.234771654
C	3.432038036	0.255999691	3.57501449
H	3.078735529	-0.691682288	3.978426093
H	3.895879708	0.063230319	2.606701926
C	2.253431274	1.182120765	3.438106115
C	1.471566484	1.232696545	2.295036733
H	1.726776369	0.619652892	1.441866185
C	0.369579879	2.077382848	2.272160291
H	-0.258438543	2.129295975	1.393013393
C	0.092213744	2.86321977	3.384556814
H	-0.748729965	3.541738392	3.402153754
C	0.923731026	2.770426183	4.486931605
H	0.760788612	3.375585266	5.367278334
C	5.205308243	1.912781015	3.899648528

H	4.562791143	2.403807011	3.169624551
H	6.049444005	1.478051532	3.363548617
C	5.666922115	2.951438778	4.891407984
C	6.990021077	3.356751863	4.978943956
H	7.740624544	2.907147536	4.343179791
C	7.326071955	4.341238012	5.904180727
H	8.351611493	4.67195309	6.001767729
C	6.329190939	4.883893666	6.703933412
H	6.547740457	5.647661047	7.437327544
C	5.030081331	4.411801454	6.555558057
H	4.222892593	4.792731022	7.16956052
N	4.709177297	0.983637162	7.228130984
N	4.402394136	0.829512214	4.538605874
N	1.973658355	1.940539247	4.512921007
N	4.704757549	3.467619334	5.670722716
O	0.75175537	5.280421989	8.094353121
N	1.533380573	5.041480899	7.279861726
O	2.026386729	2.231454865	7.500488346
H	2.082150021	1.433902141	8.14636262
P	0.83571911	-0.49883456	7.491838452
O	0.016933873	0.645278861	6.882055006
O	1.700137174	-0.008914821	8.676851162
O	0.078145632	-1.783369802	7.773113078
O	1.934123996	-0.827028272	6.263380707
H	1.132595507	1.953707239	7.140582854
H	2.505732168	-1.555999093	6.547480555

Structure: [Cu(tmpa)(HPO_4)] + NO + H_2O
Gibbs free energy: -8595.94 kcal/mol
Figure: S28

Cu	2.982412922	1.361167881	6.015627673
C	4.585007027	1.124962223	8.491940796
H	3.656643997	1.409616591	8.965163883
C	5.76618518	0.970907879	9.197180825
H	5.783533422	1.14424944	10.26371864
C	6.913635406	0.602340379	8.504217268
H	7.854959415	0.487277818	9.024854675
C	6.844915532	0.384353844	7.13369582
H	7.719210333	0.096257626	6.566905058
C	5.628794923	0.551745393	6.493130936
C	5.424984363	0.281455398	5.023741413
H	6.355156031	0.39903223	4.467731819
H	5.106816561	-0.75438438	4.908668935
C	3.544762705	0.488876822	3.42045157
H	3.423092117	-0.557980865	3.698541722
H	4.049197486	0.525546374	2.454089998
C	2.178675459	1.119628534	3.346526586
C	1.414915825	1.14494072	2.191002096
H	1.81733416	0.749323324	1.26880114

C	0.134989799	1.682005552	2.248827212
H	-0.483282168	1.710616989	1.361723153
C	-0.340524954	2.185038164	3.454661176
H	-1.330124674	2.611358344	3.537111111
C	0.480958189	2.135059435	4.567898542
H	0.162194372	2.507218386	5.530667011
C	4.891468393	2.445157651	3.951923032
H	4.042043633	3.057585195	3.654083927
H	5.490470698	2.229152996	3.065411988
C	5.723932032	3.174876517	4.969145884
C	7.103465081	3.27543335	4.832928621
H	7.59559207	2.863521469	3.962196429
C	7.834620699	3.897982697	5.838953185
H	8.910298571	3.983036698	5.760314157
C	7.161969688	4.395603222	6.945787686
H	7.688914328	4.878252651	7.757493943
C	5.776765055	4.267154851	6.98826264
H	5.213926064	4.655397718	7.830893432
N	4.531163645	0.927724975	7.169948714
N	4.349774595	1.152088736	4.478569521
N	1.711546289	1.612391339	4.505612287
N	5.074568966	3.679178549	6.02522763
O	2.681787793	4.334690189	7.737451573
N	2.618204422	3.950938966	6.645330744
O	-0.493024151	2.703268622	7.832502182
H	-0.240041313	3.275668302	8.569116577
P	1.249095603	-0.462830022	7.464397169
O	1.615354511	1.06937259	7.377857106
O	1.814111434	-1.119811251	8.704561823
O	-0.2118785	-0.691109586	7.158650651
O	2.116653324	-1.079489952	6.165649841
H	0.279448534	2.09648079	7.701208811
H	2.750276529	-1.726670268	6.508381741

References

1. Langerman, M.; Hetterscheid, D. G. H., Fast Oxygen Reduction Catalyzed by a Copper(II) Tris(2-pyridylmethyl)amine Complex through a Stepwise Mechanism. *Angew Chem Int Ed* **2019,** *58* (37), 12974-12978.
2. Krezel, A.; Bal, W., A formula for correlating pKa values determined in D2O and H2O. *J Inorg Biochem* **2004,** *98* (1), 161-6.
3. Ault, A., General Acid and General Base Catalysis. *J Chem Educ* **2007,** *84* (1), 38-39.
4. E. V., A.; D. A., D., *Modern Physical Organic Chemistry*. University Science Books: Mill Valley, California, 2006.
5. te Velde, G.; Bickelhaupt, F. M.; Baerends, E. J.; Fonseca Guerra, C.; van Gisbergen, S. J. A.; Snijders, J. G.; Ziegler, T., Chemistry with ADF. *J Comput Chem* **2001,** *22* (9), 931-967.
6. AMS 2022.1 SCM. Rüger, R.; Franchini, M.; Trnka, T.; Yakovlev, A.; van Lenthe, E.; Philipsen, P.; van Vuren, T.; Klumpers, B.; Soini, T., Eds. http://www.scm.com: Theoretical Chemistry, Vrije Universiteit, Amsterdam, The Netherlands.
7. Becke, A. D., A new mixing of Hartree–Fock and local density-functional theories. *J Chem Phys* **1993,** *98* (2), 1372-1377.
8. Lee, C.; Yang, W.; Parr, R. G., Development of the Colle-Salvetti correlation-energy formula into a functional of the electron density. *Phys Rev B* **1988,** *37* (2), 785-789.
9. Caldeweyher, E.; Ehlert, S.; Hansen, A.; Neugebauer, H.; Spicher, S.; Bannwarth, C.; Grimme, S., A generally applicable atomic-charge dependent London dispersion correction. *J Chem Phys* **2019,** *150* (15), 154122.
10. Van Lenthe, E.; Baerends, E. J., Optimized Slater-type basis sets for the elements 1-118. *J Comput Chem* **2003,** *24* (9), 1142-56.
11. Pye, C. C.; Ziegler, T., An implementation of the conductor-like screening model of solvation within the Amsterdam density functional package. *Theor Chem Acc* **1999,** *101*, 396-408.
12. Hematian, S.; Siegler, M. A.; Karlin, K. D., Heme/copper assembly mediated nitrite and nitric oxide interconversion. *J Am Chem Soc* **2012,** *134* (46), 18912-5.
